# COVID-19 false dichotomies and a comprehensive review of the evidence regarding public health, COVID-19 symptomatology, SARS-CoV-2 transmission, mask wearing, and reinfection

**DOI:** 10.1186/s12879-021-06357-4

**Published:** 2021-07-27

**Authors:** Kevin Escandón, Angela L. Rasmussen, Isaac I. Bogoch, Eleanor J. Murray, Karina Escandón, Saskia V. Popescu, Jason Kindrachuk

**Affiliations:** 1 School of Medicine, Universidad del Valle, Cali, Colombia; 2 Vaccine and Infectious Disease Organization, University of Saskatchewan, Saskatoon, Canada; 3 Georgetown Center for Global Health Science and Security, Georgetown University, Washington, DC USA; 4 Division of Infectious Diseases, University of Toronto, Toronto General Hospital, Toronto, Canada; 5 Department of Epidemiology, Boston University School of Public Health, Boston, USA; 6 Department of Anthropology, Universidad Nacional de Colombia, Bogotá, Colombia; 7 Schar School of Policy and Government, George Mason University, Fairfax, VA USA; 8 Department of Medical Microbiology and Infectious Diseases, University of Manitoba, Winnipeg, Canada

**Keywords:** COVID-19, SARS-CoV-2, Coronavirus, Pandemic, Nonpharmaceutical intervention, Harm reduction, Presymptomatic, Asymptomatic, Transmission, Outdoor, Droplet, Aerosol, Pollution, Mask, Reinfection

## Abstract

Scientists across disciplines, policymakers, and journalists have voiced frustration at the unprecedented polarization and misinformation around coronavirus disease 2019 (COVID-19) pandemic. Several false dichotomies have been used to polarize debates while oversimplifying complex issues. In this comprehensive narrative review, we deconstruct six common COVID-19 false dichotomies, address the evidence on these topics, identify insights relevant to effective pandemic responses, and highlight knowledge gaps and uncertainties. The topics of this review are: 1) Health and lives vs. economy and livelihoods, 2) Indefinite lockdown vs. unlimited reopening, 3) Symptomatic vs. asymptomatic severe acute respiratory syndrome coronavirus 2 (SARS-CoV-2) infection, 4) Droplet vs. aerosol transmission of SARS-CoV-2, 5) Masks for all vs. no masking, and 6) SARS-CoV-2 reinfection vs. no reinfection. We discuss the importance of multidisciplinary integration (health, social, and physical sciences), multilayered approaches to reducing risk (“Emmentaler cheese model”), harm reduction, smart masking, relaxation of interventions, and context-sensitive policymaking for COVID-19 response plans. We also address the challenges in understanding the broad clinical presentation of COVID-19, SARS-CoV-2 transmission, and SARS-CoV-2 reinfection. These key issues of science and public health policy have been presented as false dichotomies during the pandemic. However, they are hardly binary, simple, or uniform, and therefore should not be framed as polar extremes. We urge a nuanced understanding of the science and caution against black-or-white messaging, all-or-nothing guidance, and one-size-fits-all approaches. There is a need for meaningful public health communication and science-informed policies that recognize shades of gray, uncertainties, local context, and social determinants of health.

## Background

The coronavirus disease 2019 (COVID-19) pandemic has posed unparalleled challenges to society and upended life in a myriad of devastating ways. With over 180 million confirmed infection cases and over 3.9 million related deaths as of early July 2021 [1], severe acute respiratory syndrome coronavirus 2 (SARS-CoV-2) continues to spread globally. COVID-19 has stretched healthcare system capacity, negatively impacted mental health, exacerbated socioeconomic disparities, and devastated economies. Scientists across disciplines, policymakers, and journalists continue to operate on “Pandemic Standard Time”—struggling to meaningfully advance science, policy, and communication in real time with rapidly emerging data, while countering the unprecedented “infodemic”^1^, polarization, and politicization in pandemic response plans [3–10]. The global community is not used to seeing rapidly emerging science and changing policy, and has therefore been desperate for immediate, unambiguous answers. Naturally, intolerance of uncertainty has driven some people to fill this void with deceptive narratives [11, 12].

Misinformation and disinformation^2^ come in endless guises and spread via different mechanisms, including campaigns of persistent inaccurate beliefs and falsehoods, deceptive messages, and engagement echo chambers^3^ [13, 14]. The pandemic has brought a paper tsunami with widespread misinterpretation of both peer-reviewed research and preprints, press releases without scrutinizable data, sensationalized media reporting, and endless conspiracy theories [5, 11, 15, 16]. As a result, finding trustworthy sources of information and guidance on COVID-19 has been difficult for the public. Over the past months, logical fallacies and cognitive biases have relentlessly distracted from critical appraisal and transparent communication of the scientific evidence related to COVID-19 [17]. Confirmation bias, availability bias, motivated reasoning, the Dunning-Kruger effect, black-or-white fallacy (also known as false dilemma, false dichotomy, either/or fallacy, or false choice), straw man fallacy, ad hominem fallacy, appeal to emotion, appeal to ignorance, and appeal to authority fallacies have all run rampant across social media.

**Fig. 1:**
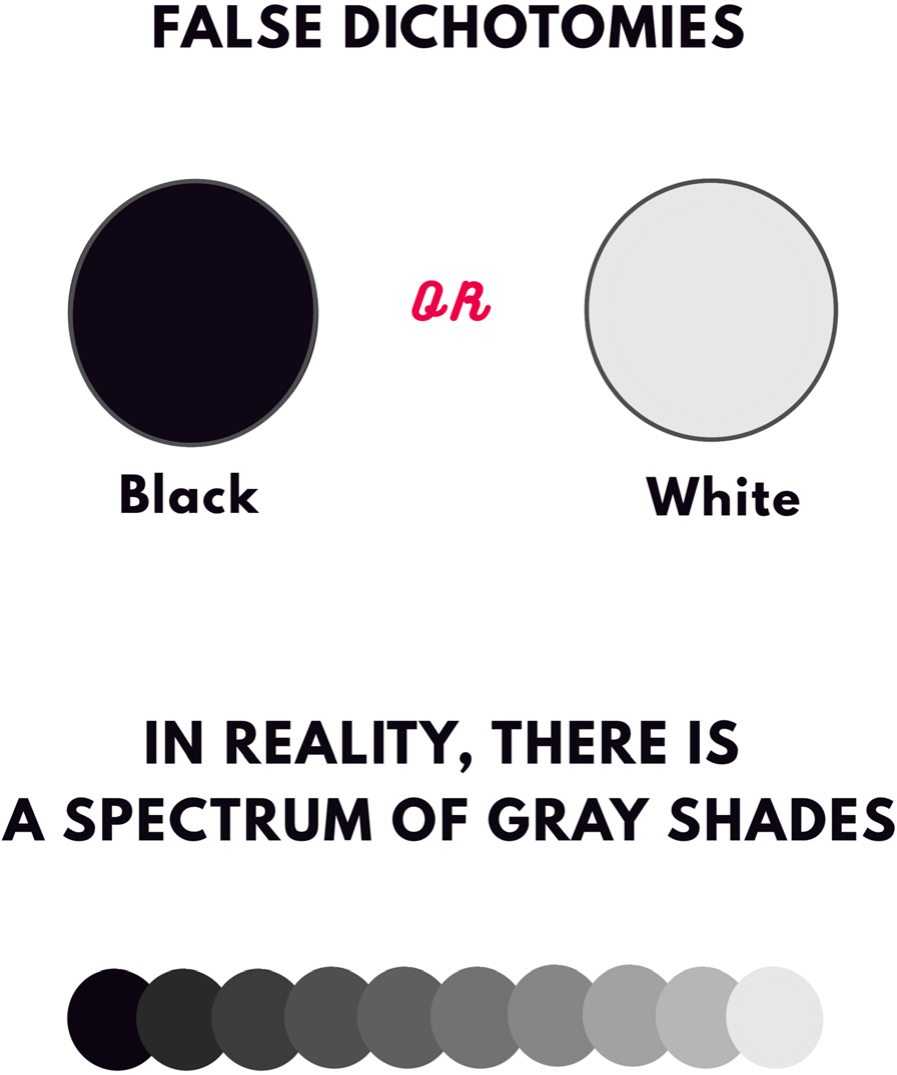
A false dichotomy is a logical fallacy that involves presenting two opposing facts, views, or options as though they were the only possibilities. The false dichotomy fallacy is often committed when someone thinks one of the two options is obviously true while the other is obviously false. In reality, many more facts, views, and options exist in between, which can be represented as a gradient of gray shades between the extremes of black and white. While reasoning in binaries may feel easier and reassuring, people unaware of false dichotomies distract from the fact that there are many alternatives

False dichotomies—statements erroneously posited as two simple, mutually exclusive options—have sparked hot debates stemming from different views on evaluating the content and sufficiency of the evidence on which to draw conclusions (Fig. 1). Opponents for either side of these conundrums see whatever data through the lens of their preconceptions, cherry-pick scientific research, and fit polarizing narratives with the perils of black-or-white messaging and reductionist frameworks. Their rigid views, fueled by misinformation, often polarize alongside the increasing certainty with which they are expressed [18, 19]. Some academics and politicians navigating the public scrutiny of COVID-19 response have been concerned that communicating scientific uncertainty undermines trustworthiness [20, 21].

**Fig. 2:**
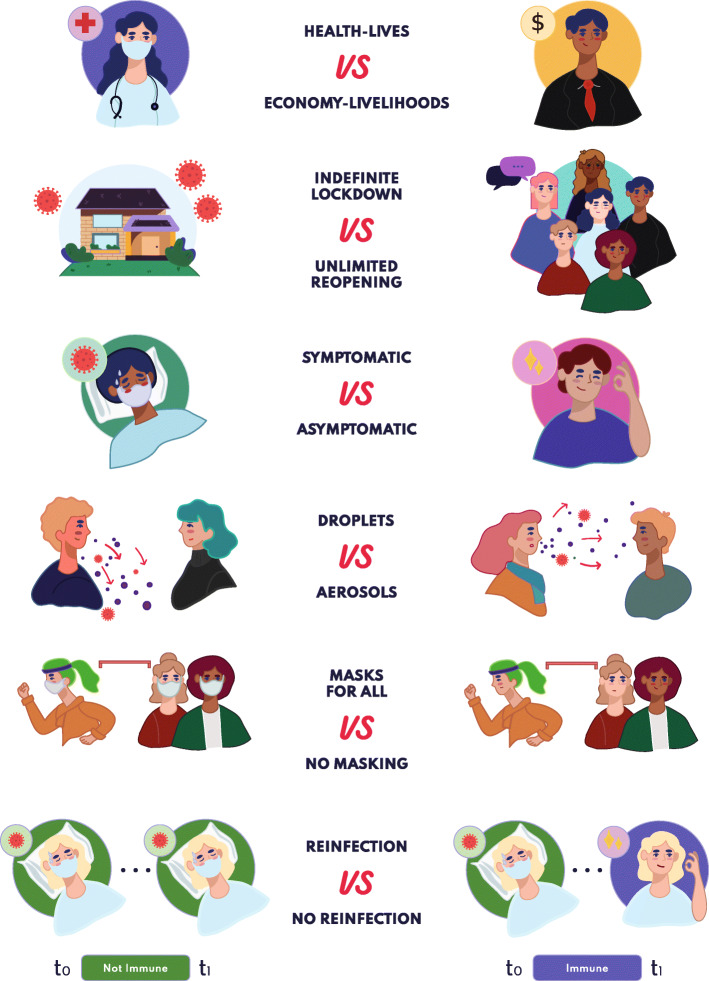
This infographic depicts the simplistic black-or-white framing and the scientific, political, and social polarization of the topics covered in this review: 1) Health and lives vs. economy and livelihoods, 2) Indefinite lockdown vs. unlimited reopening, 3) Symptomatic vs. asymptomatic SARS-CoV-2 infection, 4) Droplet vs. aerosol transmission of SARS-CoV-2, 5) Masks for all vs. no masking, and 6) SARS-CoV-2 reinfection vs. no reinfection

The COVID-19 pandemic has been riddled with false dichotomies, which have been used to shut down or polarize debates while oversimplifying complex issues and obfuscating the accompanying nuances. In this review, we aimed to deconstruct six common COVID-19-related false dichotomies (Fig. 2) by reviewing the evidence thoughtfully and thoroughly: 1) *Health and lives* vs. *economy and livelihoods*, 2) *Indefinite lockdown* vs. *unlimited reopening*, 3) *Symptomatic* vs. *asymptomatic SARS-CoV-2 infection*, 4) *Droplet* vs. *aerosol transmission of SARS-CoV-2*, 5) *Masks for all* vs. *no masking*, and 6) *SARS-CoV-2 reinfection* vs. *no reinfection*. At least three trade-offs exist at the interface of science and policy related to this pandemic: clarity-complexity (simple messages vs. conveying uncertainty), speed-quality (timely responses vs. in-depth quality assessment), and data-assumption (data availability vs. required set of assumptions) [22, 23]. Therefore, while exploring challenging and contentious topics, we make the case for a nuanced understanding of COVID-19 science, identify insights relevant to effective pandemic responses, and highlight important research gaps. We also provide examples that echo the importance of interdisciplinary integration, epistemic uncertainty in risk communication, and public health during pandemics [20, 22, 24].

A summary of key recommendations and insights is provided in the Table 1 and a lay summary is provided in the Table 2.

**Table 1:** Key recommendations and insights of the COVID-19 topics addressed in this review and recommended articles

**Science, public health, policy, uncertainty, and communication aspects of COVID-19** Recommended bibliography: [5, 9, 11, 16, 17, 24]	
• The COVID-19 pandemic is a stark reminder of ignored yet important gaps, challenges, and opportunities in scientific communication, health education, and policy implementation.	
• We need to go beyond “following the science.” The need for and interest in science provides opportunities to create better dialogue between scientists and society.	
• Conveying uncertainty does not harm public trust.	
• False dichotomies are pervasive and attractive—they offer an escape from the unsettling complexity and enduring uncertainty.	
• Debunking misinformation and discouraging black-or-white messaging, all-or-nothing guidance, and one-size-fits-all approaches are valuable endeavors.	
• Public health agencies can track COVID-19 misinformation in real time and engage communities and governments to dispel misinformation.	
**1. Health and lives vs. economy and livelihoods**	
Recommended bibliography: [8, 23, 25–27]	
• Widespread infectious disease transmission negatively impacts both health and the economy.	
• Appropriate public health strategies that reduce SARS-CoV-2 transmission safeguard both health and the economy.	
• The pandemic response must involve economic, psychological, and sociological views to ensure that lives and livelihoods are protected.	
• Public health experts, economists, social scientists, and bioethicists must work jointly to assist governments in shaping the best policies that protect the overall societal well-being.	
**2. Indefinite lockdown vs. unlimited reopening**	
Recommended bibliography: [28–40]	
• Lockdowns and other stringent public health measures bring social, psychological, and economic harm and competing health risks.	
• Regions with widespread transmission should not reopen prematurely in the absence of coordinated and robust countermeasures.	
• Multilayered NPIs are needed as part of the road maps for navigating the COVID-19 pandemic.	
• Transmission dynamics should inform policy decisions about mitigation strategies and recommendations for reopening.	
• Tailored strategies and context-sensitive policymaking fall squarely within the purview of public health and aid in honing our response to COVID-19.	
• Harm reduction, continued education, and incentivized messaging work better than shaming and blaming people for violating public health measures.	
• Encouraging outdoor activities helps mental and physical welfare, decreases the pandemic and response fatigue, and avoids risk-prone activities from going underground.	
• Policies should be constantly reassessed in the name of safety, so that their benefits always outweigh the harms.	
• Increasing vaccination rates followed by decreasing numbers of cases may allow gradual relaxation of restrictions.	
**3. Symptomatic vs. asymptomatic SARS-CoV-2 infection**	
Recommended bibliography: [41–45]	
• SARS-CoV-2 infection ranges from a complete lack of symptoms to critical disease.	
• Mild COVID-19 is the most common disease presentation.	
• Broadly, there are two types of infected individuals: symptomatic and asymptomatic. The former individuals undergo three distinct stages (usually communicated as if they were different individuals): presymptomatic, symptomatic, and postsymptomatic.	
• COVID-19 encompasses a broad clinical spectrum. Fever, cough, fatigue, and anosmia/hyposmia are the most common manifestations.	
• Testing (serial if possible), follow-up (ideally 14 days), and a thorough symptom assessment are required to avoid misclassification and truly differentiate asymptomatic individuals from presymptomatic, paucisymptomatic, and postsymptomatic individuals.	
• Differential secondary attack rates, viral shedding dynamics, and modeling estimates of contribution to transmission support greater transmission risk from symptomatic and presymptomatic individuals compared with asymptomatic individuals.	
**4. Droplet vs. aerosol transmission of SARS-CoV-2**	
Recommended bibliography: [46–53]	
• Close-contact transmission, via short-range aerosols and droplets, is the primary transmission mode of SARS-CoV-2.	
• Direct (physical) and indirect (via fomites) contact transmission play a minor role in propagating SARS-CoV-2.	
• Long-range aerosol transmission occurs under certain conditions: prolonged exposure in enclosed spaces with inadequate ventilation.	
• Epidemiological data help determine SARS-CoV-2 transmission mechanisms in real-world conditions.	
• Minimum infectious dose, particle size distribution of virus concentrations, and virus viability in particles are unknowns germane to elucidating transmission modes.	
• The term “airborne” offers no clear guidance on how to reduce exposure risk and may lead to misunderstandings of transmission or panic.	
• Public health messaging on transmission needs nuance and to be accompanied by indications on effective preventive measures.	
• Disagreement between different disciplines over SARS-CoV-2 transmission is largely related to semantics.	
**5. Masks for all vs. no masking**	
Recommended bibliography: [54–60]	
• “Smart masking” is a more accurate term than “universal masking.”	
• The case for mask wearing is strongest in high-risk scenarios such as crowded spaces, indoor venues, and unventilated places.	
• The case for mask wearing is weakest in marginal-risk scenarios such as outdoor and uncrowded environments where distancing and ventilation are possible.	
• In addition to filtration efficiency, fit, and breathability, proper and consistent wearing of masks influences their effectiveness.	
• Mask adherence is multifactorial, mediated by sociocultural and psychological factors.	
• A social norm of masking is built through well-crafted messaging plus permanent education campaigns on proper mask wearing, the right settings and times to wear a mask, and safe and legitimate exceptions to masking.	
• To encourage mask adherence and gain public acceptability, society must be transparently informed about the real-world benefits, potential downsides, and uncertainties.	
**6. SARS-CoV-2 reinfection vs. no reinfection**	
Recommended bibliography: [61–65]	
• SARS-CoV-2 reinfection remains an overall infrequent event.	
• Publication of reinfections is biased toward symptomatic cases. Asymptomatic cases are underreported.	
• Existing studies suggest that immune protection following SARS-CoV-2 infection is generally in the range of 5–12 months, though the heterogeneity of induction and durability of immune responses across individuals is acknowledged.	
• Epidemiological analyses (including clinical case history assessment) and virological data (nucleic acid amplification testing and comparative genome analysis) are needed to distinguish between reinfection, persistent viral RNA shedding, and recrudescence.	
• Additional investigations of SARS-CoV-2 damage to reproductive tissue and potential for persistence need to be determined.	

*Abbreviations*: *COVID-19* coronavirus disease 2019, *NPI* non-pharmaceutical intervention, *qRT-PCR* quantitative reverse transcriptase-polymerase chain reaction, *SARS-CoV-2* severe acute respiratory syndrome coronavirus 2

**Table 2:** Lay summary of this review

This narrative review, conducted by an international team of scientists with different backgrounds, is illustrative of the complexities of public health, policymaking, and risk communication with the public in health emergencies such as the ongoing pandemic. Here, we focus on false dichotomies, which refer to claims or positions erroneously presented as two simplistic and polarized options. While there have been many false dichotomies about SARS-CoV-2 and COVID-19, we chose six:	
False dichotomy 1. It has been said that *health* and the *economy* are on opposite poles, but this is not true. Public health and economic experts agree that supporting workers and businesses financially is key to tackling the pandemic. Sensible public health strategies that reduce the spread of the virus reduce the health and economic harms of the pandemic.	
False dichotomy 2. Discussion about response measures has pitted *indefinite lockdown* against *unlimited reopening*, but a more nuanced response is needed. While no single intervention is a silver bullet, there are many tools in our COVID-19 response kit that can be used together to further reduce risk. Response plans must be tailored to local COVID-19 levels, vaccination levels, among other issues, with a clear plan for how response measures are evaluated and implemented. Education and harm reduction are effective and sustainable approaches in the long term.	
False dichotomy 3. The dichotomy *symptomatic* vs. *asymptomatic* is simplistic. There are different stages of infection and a broad spectrum of disease manifestations in the body. About four in five infected individuals develop COVID-19 symptoms. Cases are substantially spread by infected people both when they have symptoms and when they do not. Relying exclusively on symptom-based strategies for controlling the spread of SARS-CoV-2 seems insufficient and other interventions are needed.	
False dichotomy 4. Referring to absolutes such as *droplets* vs. *aerosols* or *airborne* vs. *non-airborne* is inaccurate. Respiratory particles exist on a continuum rather than as a dichotomy. The primary transmission mode of SARS-CoV-2 is close contact with respiratory particles. Surface transmission is a minor mode of transmission. Long-range aerosol transmission occurs in specific conditions such as prolonged exposure, enclosed spaces, and inadequate ventilation.	
False dichotomy 5. *Masks* and *face coverings* are effective preventive tools but are not faultless. While mask wearing is a complex intervention, there is consistent evidence that demonstrates their effectiveness to reduce the spread of the virus. Policies must be clearly communicated and include aspects such as the scenarios where they are most useful (crowded and indoor spaces) and the importance of wearing well-fitted masks and continuing education efforts to increase adherence.	
False dichotomy 6. SARS-CoV-2 *reinfection* is rare but does occur. Reinfection can be confused with persistence of virus components in the body after infection or with reactivation of virus hidden in some body organs. Differentiating these phenomena is not easy. Evidence supports protection from reinfection for at least 6–12 months after a first infection episode. Reinfections are expected to occur only in some individuals, likely caused by fading or insufficient immunity.	
Nuance is critical in risk communication with the public and for policymaking in public health. We must recognize that there are not only two options in our understanding of COVID-19 and the public health response.	

## Methods

A comprehensive, narrative literature review of the health, social, and physical sciences was undertaken to tackle six COVID-19 dichotomies. These topics were chosen by the researchers as relevant to COVID-19 science, public health, and policy given the emerging polemics around them during 2020. Although we mention COVID-19 vaccination in several sections of this manuscript, it was not a main topic of our review given that initial versions of this manuscript were written and submitted before December 2020 (when the first real-world reports of COVID-19 vaccinations occurred). From database inception to June 3, 2021 (updated search), authors explored different databases (PubMed, Google Scholar) and preprint servers (medRxiv, bioRxiv, PsyArXiv, OSF Preprints) for all types of articles using the terms “public health,” “economy,” “lockdown,” “symptomatic,” “asymptomatic,” “presymptomatic,” “paucisymptomatic,” “severity,” “droplet,” “aerosol,” “airborne,” “mask,” “masking,” “face covering,” “reinfection,” “recrudescence,” and “immunity.” Various combinations of these terms were entered along with “COVID-19,” “SARS-CoV-2,” “2019-nCoV,” “coronavirus,” “false dichotomy,” “false dilemma,” “uncertainty,” and “risk communication.” Some authors shared known articles and gray literature otherwise not retrieved in the searches. Handsearching of articles’ bibliographies led to the identification of further studies. Because of the diverse and rapidly expanding COVID-19 research, preprints and gray literature were considered but interpreted with caution given their lack of peer-review. Included articles were mutually agreed upon by the authors. The team of authors included a mix of academics and scientists with diverse backgrounds (infectious diseases, epidemiology, virology, public health, anthropology), which allowed a science-driven and fine-grained discussion of the evidence. Insights and implications for public health were carefully analyzed.

## Main text

### False dichotomy 1: Health and lives vs. economy and livelihoods

COVID-19 response plans have often been framed in terms of a health-economy zero-sum thinking [25]. That is, public health strategies necessarily hurt a nation’s economic well-being and vice versa. The false dilemma about these two competing priorities has been extended to include civil health, for instance, the right to protest against measures such as societal lockdowns, and public health threats such as systemic racism and police brutality [54, 66–69].

There is no such dichotomy between health and the economy or between saving lives and saving livelihoods as all these concepts are intimately intertwined [23, 25]. The ongoing pandemic is both a public health and economic crisis with dreadful consequences on morbidity and mortality [26, 70]. Globally, economic contraction and growth closely mirror increases and decreases in COVID-19 cases [70]. Appropriate public health strategies that reduce SARS-CoV-2 transmission also safeguard the economy since the toll of widespread illness in workers can lead to disability and death. Aggregate data have shown that many countries that suffered severe economic hardship performed worse in protecting their population’s health from COVID-19 over the past months [71].

However, the physical and mental health effects and the profound socioeconomic impact of COVID-19 and the related countermeasures must not be overlooked [8, 27, 72]. Health disparities driven by existing socioeconomic and racial/ethnic inequities are prevailing challenges during this pandemic [8, 25, 27, 28, 73–75]. Disadvantaged, rural, low-paid, and non-salaried individuals, blue-collar workers, informal workers, daily-wage earners, migrants, and people with mental health and addiction problems are more likely to be harmed by both the pandemic and the response. Healthcare and socioeconomic disparities differentially impact the capacity of vulnerable populations to engage in physical distancing responses [76].

Therefore, public health experts, economists, social scientists, and bioethicists must work jointly to assist governments in developing interventions that protect the overall societal well-being [8, 23, 27]. For example, governments should mitigate the wider impact of COVID-19 by considering universal healthcare coverage, basic income protection and payment freezes on rents and loans for individuals affected by lockdowns and interpersonal physical distancing measures, paid sick leave and paid quarantine leave for infected and exposed workers, stimulus payments for high-risk and essential^4^ workers, and mental health support. The International Monetary Fund also highlights the importance of identifying and supporting workers in informal employment sectors [70]. A clause like “we are going into lockdown” should be followed by a second clause like “and this is how we are going to support you during this time” [77]. The COVID-19 pandemic has painfully revealed the importance of caring for vulnerable populations, ensuring food and medicine supply chains, keeping non-COVID-19-related healthcare services, generating employment, adapting businesses, and addressing children deprived of learning and subjected to psychological distress caused by the pandemic [8, 27].

### False dichotomy 2: Indefinite lockdown vs. unlimited reopening

#### Stringent public health measures vs. natural herd immunity

Early in an infectious disease epidemic, public health responses mainly rest on our capacity to separate infectious, exposed, and susceptible individuals. Yet, inconsistencies in pandemic preparedness plans and delays in implementing robust testing and contact tracing prohibited reliance on the isolation of infectious individuals and quarantine of exposed individuals to bring SARS-CoV-2 under control [23]. Given the progression to community transmission (where numerous cases are not linkable to transmission chains or clusters), many governments enacted lockdowns^5^, stay-at-home orders, travel bans, curfews, and closing of workplaces, schools, and other community gathering spaces such as gyms and entertainment venues [25].

Such blunt measures were deployed by governments during times of unabated community transmission and high surges in cases [23]. Many public health experts viewed them as stopgap tools needed in unprepared regions with widespread virus transmission to restrict SARS-CoV-2 transmission chains during the first moments of the pandemic, while the test-trace-isolate infrastructure, personal protective equipment (PPE) supplies, and hospital capacity were scaled up and strengthened [23, 78–81]. However, because of inconsistent messaging about the purpose of lockdowns and the uncertain duration of the pandemic and response, many people believed COVID-19 was no longer a threat when lockdowns were lifted [23].

Alternative approaches were proposed when the second wave emerged in many countries. In particular, the Great Barrington Declaration (GBD) signatories proposed a dangerous and impractical approach that relied on focused protection of “high-risk” individuals while allowing uncontrolled viral transmission among “low-risk” individuals [82–84]. They argued that such a strategy would eventually lead to natural herd immunity at the population level, but this only reflected a misunderstanding of virology and immunology principles and management of public health emergencies [85–87]. The GBD strategy turned out to be an illusory way to rush back to normality, which understandably gained community and government supporters as a result of public discontent over lockdowns and diminishing trust in public health agencies [82–84]. Their rhetoric stoked, if not created, a false choice between total lockdown and a wholesale return to pre-pandemic life [84].

#### The harmful effect of stringent public health measures

Many models designed to predict the benefits of public health interventions ignored the potential harms [8]. This occurred because the earliest research on COVID-19 predominantly focused on the immediate and direct consequences of interventions such as reducing SARS-CoV-2 transmission. Currently, a growing number of reports substantiate the socioeconomic and psychological impact of both the COVID-19 pandemic and response, in addition to competing health risks [8, 27, 88].

The unintended consequences of several stringent public health interventions are massive and risk turning one public health crisis into many others [8, 23, 27, 89]. Stringent measures deeply aggravate hardship for the poor and those whose economy depends on daily informal work. Unfortunately, amid the pandemic, lockdowns and mobility restrictions were implemented globally and for extended periods, without appropriate communication to allow for public health preparedness. Furthermore, social, mental, and financial support to alleviate the negative impact of lockdowns was not provided to citizens in many countries. As a result, these unmitigated repercussions fueled calls and marches to demand the lift of lockdowns.

Adverse effects of stringent public health measures include financial downturn, unemployment, mental illness, child abuse, domestic violence, hunger, and disruption to education, child development, immunization programs, contraception, and family planning [8, 27, 89–95]. Discontinuation of clinical services and prevention efforts regarding chronic non-communicable diseases [96, 97] and infectious diseases other than COVID-19 (e.g., HIV infection, tuberculosis, malaria) has been reported [88, 98, 99]. Because the current pandemic is risking decades of progress in other infectious diseases and existing public health threats, strengthening of healthcare systems and a reassessment of global health funding and policies are urgently needed [88].

#### Finding a balance between lockdowns and unlimited reopening

In the presence of widespread community transmission, regions reopening prematurely without a coordinated, robust plan will face COVID-19 resurgence. This can force societies to go back to general or targeted lockdowns after uncontrolled outbreaks, as repetitively happened in countries that underwent staggering rises in COVID-19 cases, hospitalizations, and deaths following unfettered reopening. Robust policies with continued monitoring, non-pharmaceutical interventions (NPIs), and plans to avert overwhelming healthcare systems are critical from the beginning of an epidemic to avoid catastrophic scenarios. Alert level systems, informed by the level of community transmission and impact of COVID-19, are useful tools for escalating or de-escalating restrictions based on their impact and the response goal.

Rather than posing an all-or-nothing dilemma, striking a balance between continuing indefinite shutdowns and returning to pre-COVID-19 normality is needed. A stepwise, cautious lifting of lockdowns and loosening of other restrictions that help economies and social life continue are possible through the implementation of multipronged NPIs with lesser economic, societal, and quality-of-life costs [8, 29, 30, 80]. Tens of NPIs have been described in the pre-COVID-19 literature and have been reassessed during this pandemic as countries have tailored their response plans. Examples of NPIs are physical distancing, mask wearing (discussed in section 5), natural or mechanical ventilation of indoor spaces, limiting non-essential social contact, avoiding crowded indoor spaces, hand hygiene, respiratory etiquette^6^, avoiding touching the face, cleaning and disinfection of surfaces, air filtration, robust testing (with short turnaround times), rigorous contact tracing, isolation of infected individuals, quarantine of close contacts, mass gathering bans, travel restrictions (e.g., entry and exit restrictions, travel advice and warnings), temperature and health checks, staggered work shifts, rotational groups, telework initiatives, and redesign of living, teaching, and working environments to prevent crowding [30–32, 100, 101].

During 2020, several regional economies were able to progressively resume to varying extents and worked to overcome logistical hurdles and implement combinations of preventive measures. However, controlling the spread of SARS-CoV-2 has proven challenging. Since December 2020, when the first reports of COVID-19 vaccinations outside clinical trials were published [102], the world has gained hope and seen the tangible benefits of vaccination. COVID-19 vaccines are a ground-breaking achievement that will help to end the pandemic [33]. However, the world will require complementary NPIs as long as a large share of the population is not vaccinated. The current global situation of more transmissible genetic variants of SARS-CoV-2 has raised concerns, but the remarkably high effectiveness of available vaccines is encouraging ([33, 103], Escandón K., Flocco G., Hodcroft E.B. et al., unpublished data). No effective SARS-CoV-2 antiviral is currently available.

#### Multilayered prevention and additive risk reduction

**Fig. 3:**
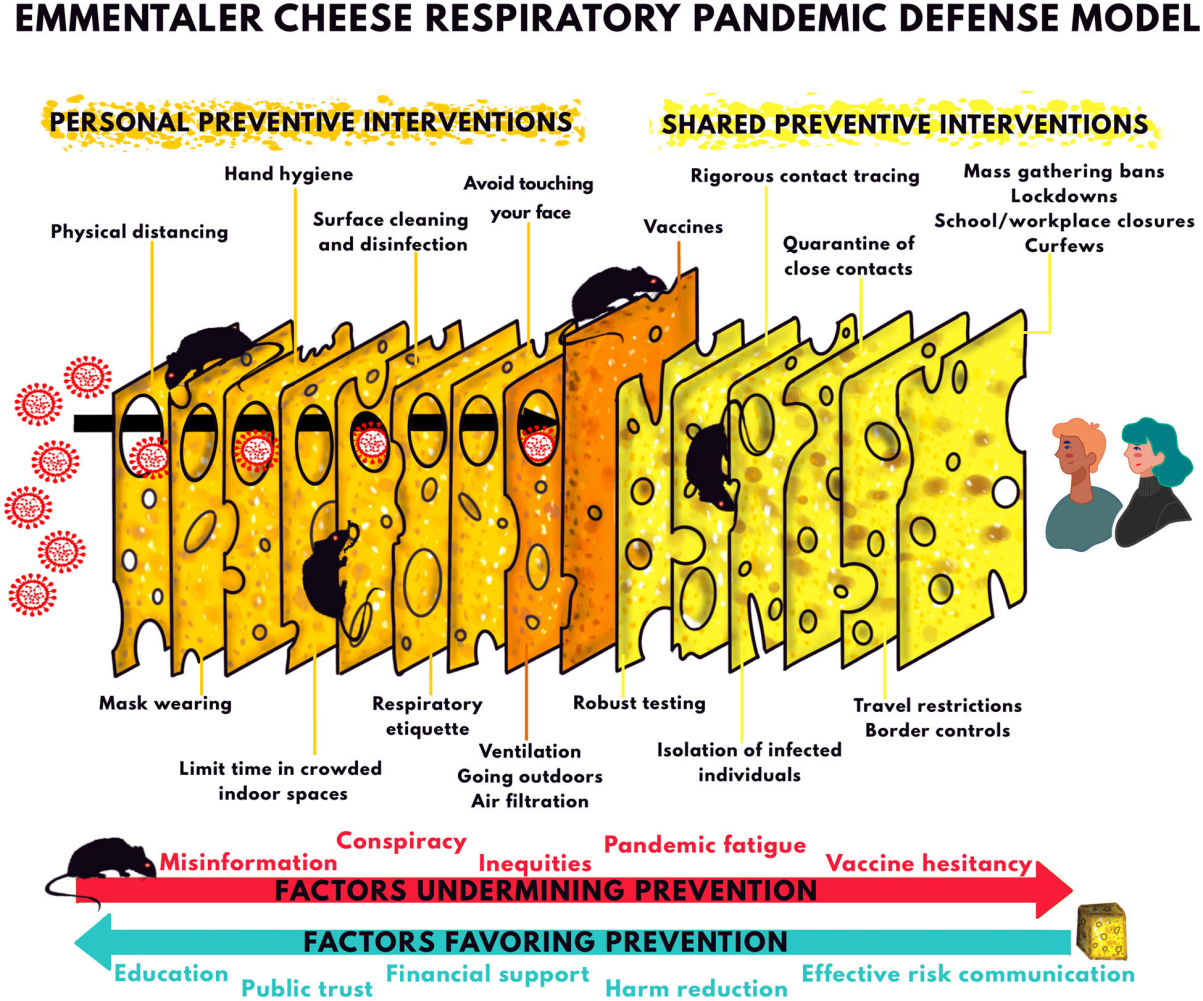
The “Swiss cheese model” of accident causation (more accurately called Emmental or Emmentaler cheese model [104]) originated with James T. Reason and Rob Lee in the 1990s (and was potentially influenced by other researchers) [105, 106, 107]. As applied to COVID-19 [34, 108, 109, 110, 111], this model recognizes the additive success of using multiple preventive interventions to reduce the risk of SARS-CoV-2 infection. No single slice of cheese (public health strategy) is perfect or sufficient at preventing the spread of SARS-CoV-2. Each slice has holes (inherent weaknesses or limitations) with variable number, size, and location over circumstances or time, which may allow viral transmission. SARS-CoV-2 infection occurs when multiple holes happen to align at the same time permitting a trajectory of successful transmission. When several interventions are used together and consistently and properly, the weaknesses in any one of them should be offset by the strengths of another. The preventive interventions can be broken into personal and shared, although some interventions may be both. The order of the slices and holes in the illustration are not reflective of the degree of effectiveness of the interventions, given that the scenarios of transmission are variable and complex. The black rats eating the slices of cheese represent factors undermining prevention efforts while the extra cheese represents a source of factors and opportunities favoring prevention efforts. This infographic was designed for this manuscript and was inspired by previous illustrations by the Cleveland Clinic [108], Sketchplanations [109], and virologist Ian M. Mackay, who proposed the Swiss Cheese Respiratory Pandemic Defense [34, 110]

The additive nature of risk reduction poses challenges for science communication. Education on multilayered prevention and public-facing communication efforts are negatively impacted by false dichotomies that confuse, distract, or give the appearance that only certain layers of risk reduction are important. The Emmentaler Cheese^7^ Respiratory Pandemic Defense Model, based on the “Swiss cheese model” for understanding system accidents and improving safety management in healthcare, engineering, and aviation fields [105–107], is useful to understand the importance of multilayered prevention in COVID-19 response through personal and shared public health interventions (Fig. 3) [34, 108–111]. No single cheese slice or layer of defense (risk-reducing intervention) is sufficient and perfectly protective (100% effective), but a suite of personal and shared interventions forms a robust prevention strategy [30, 31, 101]. Importantly, there are systemic factors that may contribute toward either risk reduction or risk increase of SARS-CoV-2 transmission and infection, by favoring or undermining the uptake and compliance of strategies. For instance, while misinformation and socioeconomic inequities erode trust in public health and compliance with interventions, effective risk communication and harm reduction approaches promote awareness and sensible use of NPIs to mitigate both the risk of infection and pandemic fatigue. Of note, this model is not intended to explain the complex factors involved in SARS-CoV-2 transmission or suggest a hierarchy of effectiveness of the preventive measures. This limitation does not detract from its usefulness as a means to communicate multilayered prevention and additive risk reduction. Pandemic response plans rely on the healthcare infrastructure, technical expertise, and political will across countries and regions. The combination of measures deployed will therefore vary substantially depending on dedicated resources, community transmission levels, and a close examination of their costs and benefits. Measures may have varying degrees of effectiveness and different costs.

#### Transmission dynamics and risk assessment

Transmission dynamics should inform policy decisions about risk mitigation strategies and recommendations for safer socializing and reopening [28, 46]. Targeted policies should consider the scenarios where transmission is more likely. Contact tracing provides valuable information about transmission dynamics. SARS-CoV-2 infection risk depends on physical proximity, location, type of activity, and duration of contact [28], with transmission dominated by superspreading events (SSEs) or contexts^8^, crowded spaces, indoor venues, and unventilated places. There is solid evidence on the clustering and superspreading (overdispersion^9^) potential of SARS-CoV-2, which suggests that a small part of cases (5%–29%) is responsible for the majority of transmission events (~80%) [112–114, 116, 117]. The transmission heterogeneity or superspreading of SARS-CoV-2 is both the Achilles’ heel and the cornerstone of COVID-19 control efforts [35, 112, 118].

Higher-risk scenarios include residential congregate settings (e.g., nursing homes, homeless shelters, correctional facilities, university dormitories), high-density workplaces (e.g., meat and poultry processing plants, warehouses, manufacturing and distribution companies), public transportation, family/friend/work gatherings in indoor settings, mass gatherings (especially indoors), entertainment and leisure venues, religious events, and any other unventilated places [35–37, 113, 119, 120]. All these scenarios are relevant to risk communication and mitigation efforts. Conversely, low-risk settings and activities, such as outdoor and uncrowded environments where physical distancing and ventilation may be ensured, do not drive SARS-CoV-2 transmission in significant ways.

Education and consistent risk communication with the public are critical for an effective pandemic response. Public health agencies and policymakers can educate people about the spectrum of risk and how to differentiate between higher-risk and lower-risk activities [28]. A notable example of clear and effective public health messaging is that of Japan, consisting in avoiding the “3 Cs” driving transmission—closed spaces (with poor ventilation), crowded places, and close-contact settings (such as face-to-face conversations) [121]. On the other hand, misguided policies can undermine public trust and jeopardize engagement in effective public health measures. Inaccurate accounts of transmission can lead to harmful policies and may cause individuals to fixate on inefficient or unnecessary interventions [33]. Amid the pandemic, many outdoor activities and settings (e.g., parks, beaches, hiking trails, playgrounds, skiing spots, other outdoor recreational spaces) have been discouraged or even prohibited [122–126]. In 2020, it was common that some politicians and the media called out seemingly dangerous behavior by spotlighting people frolicking on beaches, picnicking in parks, or participating in protests [66, 127, 128]. Also, overcautious people picked some studies and media reports to warn against going outdoors and spark alarm about walkers, runners, and cyclists spreading the virus via a slipstream effect over long distances [129–132]. These claims were mainly based on studies with no virological considerations and limited environmental assumptions [129, 130]. All these aspects greatly influence viral transmission (addressed in section 4).

#### Harm reduction and the low risk of outdoor transmission

Since long-term restrictive measures come with enormous collateral damage and real-world conditions lead individuals to take some risks, the way forward is to advocate a harm reduction approach instead of social abstinence-only policy [29, 38, 77]. Applied to COVID-19, harm reduction entails enhancing awareness about SARS-CoV-2 transmission and infection risk mitigation, self-assessment of risk related to personal activities, and engagement through alternatives of safer socializing. Although finding balance in the response plans is not an easy task, harm reduction is a sustainable and realistic strategy and a way of negotiating a middle ground. Allowing people to make their own compromises and informed judgments make harm reduction an ethically correct approach that enhances community engagement and trust [30, 77]. In contrast, COVID-19 absolutism^10^ is not a viable or reasonable strategy [133].

Scolding and moral outrage are counterproductive to the COVID-19 response and can perpetuate stigma. Casting shame and blame on people violating public health measures should be avoided [29, 134, 135]. Incentivized messaging works better than “pandemic shaming” and condescending messaging (e.g., #covidiots, #dontbestupid, #letthemdie) [77, 134–136]. Effective risk communication and education campaigns are therefore central to harm reduction. Harm reduction strategies may also encourage infected individuals to self-isolate and their contacts to self-quarantine in order to prevent further transmission [28].

Outdoor activities are arguably one of the mainstays of COVID-19 harm reduction by supporting mental and physical welfare and alleviating the pandemic response fatigue^11^, while curtailing infection risk [29, 33, 38, 39, 122, 123, 137]. The costs of not encouraging outdoor activities should not be overlooked. Policies that prohibit outdoor activities^12^ may result in the movement of behaviors that are objectively safe outdoors to less-safe indoor settings [29, 134]. Outdoor activities are unlikely to drive SARS-CoV-2 transmission substantially because of the higher viral particle dispersion, reduced person-to-person contact, and external environmental factors [40, 138, 139]. The scarce instances of outdoor SARS-CoV-2 transmission suggest an extremely low risk of transmission [40, 138, 139]. Four studies have found that 0.03% [36], 0.11% [140], 0.87% [119], and 2.3% [37] of reported SARS-CoV-2 cases seem to have occurred in outdoor settings. One study reported that 3.7% of cases were acquired outdoors; however, the definition of indoor setting was poorly limited to mass accommodation and residential facilities, with all other categories defined as strict outdoor settings [141]. Other studies reported that 5.3% of SARS-CoV-2 cases were associated with outdoor environments or mixed environments (with indoor and outdoor components) [37], and 9.7% of cases were related to partly outdoor occupations (construction laborers and tour guides with 4.85 percentage points each) [142]. In a preprint study, both the odds of overall transmission and the odds of SSEs were much lower outdoors (18.7-fold and 32.6-fold, respectively) [143]. A study among attendees of an overnight camp provided little information about the risk of outdoors vs. indoors, but the fact that the outbreak was clustered by cabin assignments suggests a high likelihood of transmission in indoor spaces during overnight cabin stays rather than during outdoor activities during the day [144].

A systematic review on outdoor transmission reported finding <10% of SARS-CoV-2 cases occurring outdoors [138]. However, the real figure of outdoor SARS-CoV-2 infection proportion is certainly lower. In the study by Lan et al. [142], the cases in construction laborers and tour guides may have occurred in indoor locations. Likewise, in three publications based on a crowdsourced database led by the London School of Hygiene and Tropical Medicine [37, 119, 120], there may be an overestimation since construction workers could have been infected indoors (the most updated article is the one published by Lakha et al [37]). The unreviewed paper by Nishiura et al., though widely cited, warrants caution given the lack of descriptive detail and raw data [143]. Considering the studies cited here and the potential overestimation due to misclassification of setting, it seems likely that the risk of outdoor transmission is <1%. In summary, despite the high heterogeneity in the studies describing outdoor SARS-CoV-2 transmission (i.e., non-uniformity of outdoor definition, non-systematic testing of occupational groups, reporting bias, misclassification of outdoor exposure locations) and the difficulties in linking an infection to a specific exposure or transmission source, the existing evidence consistently highlights outdoor transmission as a negligible driver of the pandemic, compared with indoor transmission [40, 138, 139].

Mass gatherings^13^ deserve discussion. The risk in mass gatherings is expected to come from unplanned, informal, unregulated, and unmitigated events or activities that lack consideration of risk mitigation measures [40, 139]. Several factors influence transmission in these settings [40, 139, 146, 147]: 1) the environment (i.e., outdoor or indoor), since it contributes ventilation; 2) the geographic scope of the event and the extent to which vulnerable or susceptible individuals may be present (e.g., local vs. international event, attendee ages); 3) event-specific behaviors that influence transmission (e.g., communal travel, indoor congregation in other venues, congregate accommodations, face-to-face vs. side-to-side arrangement, loud conversations, shouting, singing); 4) gathering size, density, duration, and attendee circulation; 5) preparedness to conduct rapid contact tracing in the event of an outbreak; and 6) the multilayered prevention approach adopted. In addition, the underlying transmission levels or infection rates in a community are likely to influence the impact of either permitting or prohibiting mass gatherings. As for outdoor gatherings, upon consideration of crowd density, size, duration, circulation, and preventive interventions, public health officials may balance and mitigate risk across different factors mentioned [40, 139]. That is, an increase in one risk factor may be offset or mitigated by decreasing other risk factors. Therefore, all mass gatherings will not generate equal risks of SARS-CoV-2 transmission and will not need homogenous mitigations [148]. Since mass gatherings may have sociocultural, economic, physical, and mental health implications, it is critical to consider the societal needs. For instance, Black Lives Matter protests in the USA were illustrative of the trade-offs offered by harm reduction. No evidence supported a growth in COVID-19 cases following the protests [66, 68], which may have been due to the outdoor environment and compensating behaviors such as the observed increase in stay-at-home and masking compliance during the protests.

#### The need for reassessing health policies in the name of safety

Successful COVID-19 experiences of some countries have encouraged others to incorporate new elements into their plans and reassess existing elements that may be causing harm or may be ineffective. As this pandemic is not over, it is necessary to constantly revisit policies in the name of safety, so that their benefits always outweigh the harms [33]. The negative impact of blanket measures such as shutdowns and school/workplace closures is expected to be worse in the poorest regions [8, 27, 149], making the quintessential case for interventions targeted to the local context rather than generalized closures. In general, a combination of context-sensitive measures should be favored over blanket measures.

One topic that has caused intense debate is the closure of schools. International organisms and public health advocates have warned about the negative impact on children’s learning, mental well-being, social support, nutrition, and safety [33, 150]. School closures should be the last resort in the COVID-19 response that countries and states pick and rely on. Evidence has emerged regarding limited SARS-CoV-2 spread within schools when sufficient preventive measures are in place, which has encouraged school reopening initiatives [151–153]. Of note, in-person schooling plans in the setting of high community transmission must include well-implemented alternative school-based mitigation strategies to not risk accelerating the pandemic [153, 154]. These considerations may allow schools to safely reopen and stay open.

Other interventions that should be de-emphasized given their limited or relatively low utility are excessive surface cleaning and disinfection, temperature checks (particularly with inaccurate techniques), and some travel-related measures [30, 33, 155].

#### Relaxation of NPIs in the context of robust vaccination

Increasing vaccination rollout followed by decreasing local infection rates may allow the progressive easing of restrictions [33]. Gradual relaxation of interventions is essential to gain and recover trust in public health. This must consider the local impact of guidance and social disparities in addition to metrics of vaccination status and COVID-19 deaths. For instance, the US Centers for Disease Control and Prevention (CDC) issued on March 8, 2021 a set of public health recommendations, where they acknowledged that fully vaccinated people (those with ≥2 weeks after receiving a full vaccination scheme) could visit other fully vaccinated people indoors without NPIs, visit with unvaccinated people at low risk for severe COVID-19 without NPIs, and refrain from testing and quarantine following a known exposure if asymptomatic [156]. Recently, in May 2021, these guidelines were updated to reflect the successful vaccination rollout and the subsequent drop in cases and deaths in the USA [157, 158]. As of writing, the CDC is supporting that fully vaccinated people no longer wear a mask or physically distance in any setting in the country, except where required by local regulations and workplace guidance, and refrain from quarantine and testing following a known exposure, if asymptomatic, with some exceptions for specific settings. Thus far, the effects of such policy decisions are illustrative of positive reinforcement in the context of efficient vaccine rollout. Publicly available data suggest that lifting mask mandates can allow a continued decrease in cases while leading to an increase in vaccine shots [159]. Vaccines and the subsequent relaxation of NPIs are contexts where messaging hope (since it is grounded in reality) has proven its value.

Another example that has been overlooked is the possibility of relaxing visitor restrictions in hospitals, provided that visitors assess their own risk and take precautions (e.g., vaccination, use of PPE, hospital screening) [160, 161]. Given the endless benefits of visitors in patient-centered care, some authors have called for more accommodating hospital policies with careful use of PPE and monitoring, even before COVID-19 vaccination was made available [160]. Currently, in places where vaccination rates are high, COVID-19 cases and deaths are decreasing and non-essential community indoor venues are open. In this context, keeping inflexible no-visitor policies in hospitals makes no sense [161].

#### Public support and the need for an explicit pandemic response goal

One of the biggest challenges in pandemic response for many countries has been the lack of a clearly articulated goal. In infectious disease response, the potential goals are control at acceptable levels, (local) elimination^14^, or (global) eradication^15^ [162]. Few countries, including New Zealand, Taiwan, Australia, China, and Vietnam, have articulated a goal of elimination as their official pandemic policy [163]. This goal has spurred leadership to enact stringent and robust COVID-19 responses including quarantine, contact tracing, and travel restrictions, among other measures, and a clear target goal appears to have aided in buy-in from the public. As a result, some countries and regions have achieved elimination and resumed pre-pandemic life, with only intermittent response to imported cases needed.

However, “Zero COVID-19” Alliance, an initiative by vocal proponents of the goal of elimination, lists several inconsistent goals, for example aiming for zero cases, hospitalizations, and deaths, stopping the spread of SARS-CoV-2 regionally, and having a world without COVID-19 (i.e., eradication) [165]. Further, critiques of elimination goals point to several shared features of successful countries. In particular, many countries that have achieved elimination of COVID-19 are island nations that deployed early, widespread, and stringent mitigation strategies. Indeed, elimination of COVID-19 appears to require an optimal surveillance system and extreme measures and may not be feasible in countries where border control is more challenging [163].

Eradication of COVID-19 is unlikely. Only two infectious diseases have ever been eradicated (smallpox and the animal disease rinderpest) [162]. Without wide-scale coordination and consensus for eradication, elimination will continue to require intensive case surveillance, quarantine or testing of travelers, and intermittent reinstatement of control measures. Despite this, local and national governments can engage in dialogue about their COVID-19 goals [163]. When elimination is not the target, control of infection below acceptable levels is the main alternative. However, the level of infection that is deemed “acceptable” is not a scientific or objective fact—rather, it is a sociological and political objective. The public must be provided with information about the target levels of infection and allowed to weigh in on whether this level is acceptable to them in order to ensure acceptance of, and cooperation with, required restrictions and interventions.

In 2020, in the absence of vaccines, COVID-19 elimination was unrealistic for most countries. Nevertheless, COVID-19 elimination is now more feasible with approved vaccines. Vaccination can purposefully lower the threshold to achieve elimination by generating low incidence infection rates and high population immunity [163, 164], without the need for stringent NPIs. Unfortunately, even with vaccines, elimination is an unrealistic goal for countries suffering from a lack of resources, political commitment, public engagement, and coordinated response plans. Vaccine inequity further complicates the situation.

### False dichotomy 3: Symptomatic vs. asymptomatic SARS-CoV-2 infection

Since the beginning of the pandemic, there has been confusion and debate over the clinical presentation of COVID-19 and asymptomatic SARS-CoV-2 infection (ASI). It is necessary to look beyond readily observable symptomatic individuals and those completely asymptomatic yet presumed to be infected. Reviewing the terminology needed to differentiate infected individuals and the infection stages is therefore the right first step before diving into the complexities between the poles of this false dichotomy.

#### Terminology: asymptomatic, symptomatic, presymptomatic, postsymptomatic, and paucisymptomatic

**Fig. 4:**
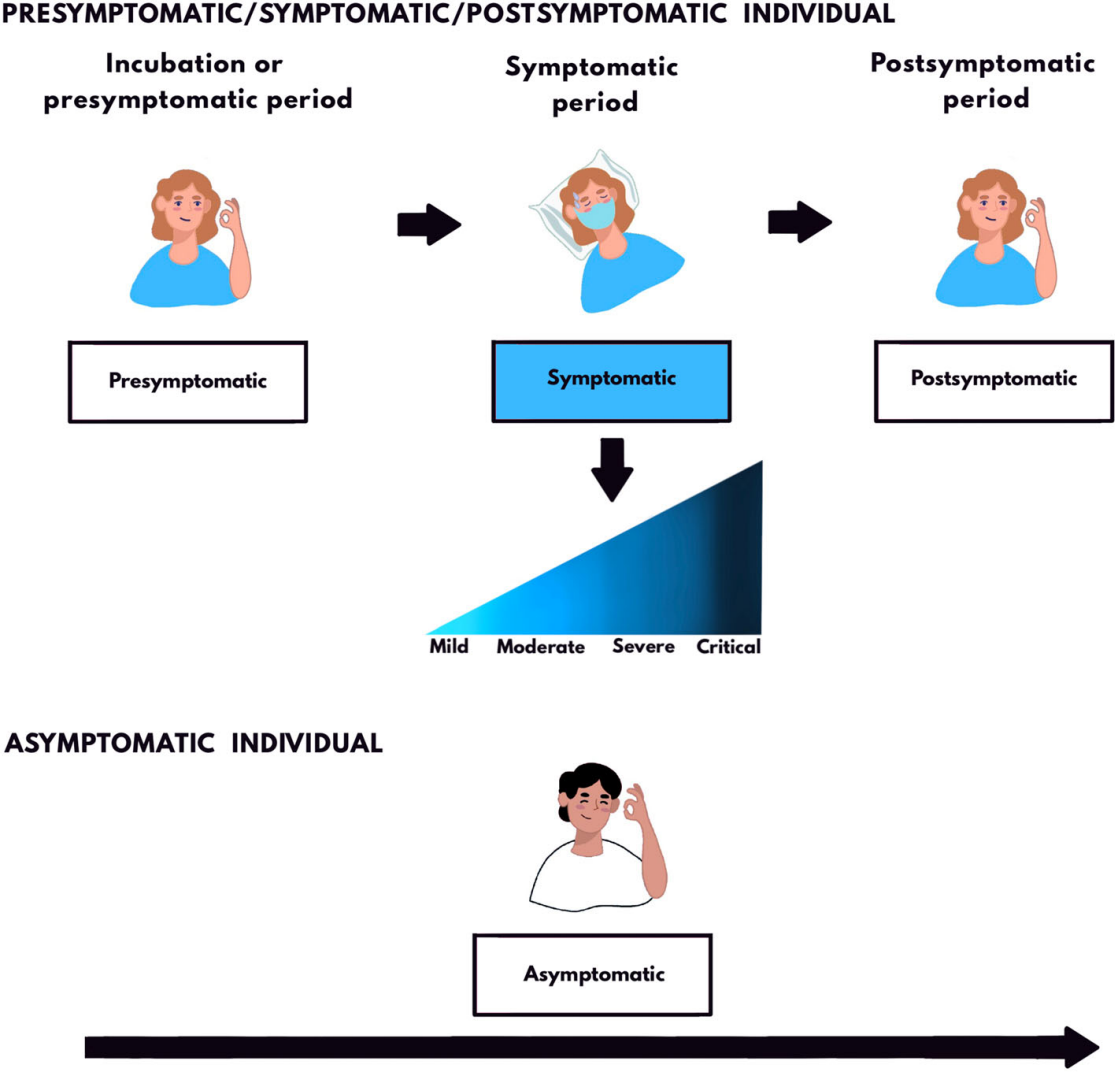
There are two types of SARS-CoV-2-infected individuals: those that develop symptoms at some point (symptomatic in a broad sense, ~75%–84%) and those that never develop symptoms (asymptomatic, ~16%–25%). The former individuals undergo three stages of infection: presymptomatic (where viral RNA is detectable but there are no symptoms), symptomatic (in a strict sense), and postsymptomatic (symptoms are gone but viral RNA is still detectable). They are often referred to as presymptomatic, symptomatic, or postsymptomatic individuals. These stages have distinct implications for transmission. Since all SARS-CoV-2-infected individuals are initially symptomless, testing, follow-up, and a thorough symptom assessment are required to truly differentiate asymptomatic from presymptomatic, paucisymptomatic (individuals experiencing mild or few symptoms), and postsymptomatic infection

Asymptomatic individuals experience no symptoms throughout the entire course of infection [41]. The remaining individuals, referred to as symptomatic (in its broad sense), initially demonstrate no symptoms during the incubation period^16^ (presymptomatic stage), then develop symptoms (symptomatic stage), and later become symptomless again during convalescence (postsymptomatic stage). As illustrated in Fig. 4, the terms presymptomatic, symptomatic (in its strict sense), and postsymptomatic refer to different stages of infection in the same infected individual rather than to different infected individuals. While classification into these three categories is only possible through retrospective and prospective symptom assessment, the stage is defined at the time of first positive test or diagnosis (i.e., presymptomatic individuals have not yet developed symptoms at the time of testing and postsymptomatic individuals experienced prior symptoms). Among individuals with active symptoms, paucisymptomatic (sometimes referred to as oligosymptomatic) individuals are regarded as those who experience mild or few symptoms attributable to the infection. A population-based study arbitrarily defined paucisymptomatic individuals as those having one or two COVID-19 symptoms (except for anosmia and ageusia) [167].

#### COVID-19 clinical presentation

SARS-CoV-2 infection can present with a broad spectrum of clinical manifestations and disease severity. COVID-19 symptoms and signs include fever, cough, fatigue, chemosensory dysfunction (i.e., anosmia/hyposmia and ageusia/hypogeusia/dysgeusia), dyspnea, headache, gastrointestinal symptoms, among others [168, 169]. COVID-19 can be categorized into mild, moderate, severe, and critical [170, 171]. COVID-19 is mild in most individuals, with no evidence of viral pneumonia or hypoxia and with symptoms that are not significant enough to seek medical attention [172]. Patients with moderate COVID-19 have evidence of non-severe pneumonia and therefore may present with dyspnea but not hypoxemia [172]. Severe COVID-19 indicates pneumonia in the presence of marked tachypnea, hypoxemia, and/or progression of lung infiltrates in chest imaging [170, 171]. Patients with critical COVID-19 are those who progress to complications such as respiratory failure, shock, and multiple organ dysfunction, often accompanied by high mortality [170, 171]. Few studies have estimated the proportions of COVID-19 across the entire spectrum of severity using the ordinal classification above. Among a cohort that included over 44,000 confirmed COVID-19 cases from China (individuals of all ages), 81% of patients developed mild or moderate COVID-19, 14% developed severe COVID-19, and 5% developed critical COVID-19 [173]. All fatal outcomes were consistently reported among critical cases. The case fatality rate was 2.3% (49% of critical cases).

Some SARS-CoV-2-infected individuals experience persistent symptoms following recovery of acute illness, which is frequently referred to as post-acute sequelae of COVID-19 (PASC) or “long COVID-19” [174–179]. Many features of PASC resemble chronic fatigue syndrome/myalgic encephalomyelitis [175, 180]. The most common symptoms of PASC are fatigue, neuropsychiatric symptoms (e.g., “brain fog,” headache, sleep difficulties, attention disorder), hair loss, dyspnea, and persistent smell or taste impairment [174, 175, 179]. There are also rare reports of hyperinflammatory syndromes (e.g., multisystem inflammatory syndrome in children [MIS-C] and adults [MIS-A] [181–183]), potentially associated with cytokine storm/release syndrome [184].

#### The proportion of asymptomatic SARS-CoV-2-infected individuals

The true occurrence of ASI is difficult to evaluate. The percentage of truly asymptomatic SARS-CoV-2-infected individuals has been variably estimated from less than 1% to as high as 96% [41, 185, 186]. Earlier reviews and opinion pieces reported wide ASI ranges (1%–88%) [187–189]. Others concluded that the overall ASI was approximately 40%–45% [186] or even conjectured that rising trends (e.g., 81%–95%) of ASI in some populations were the result of mask wearing [190–192] (further discussed in section 5). However, several concerns with these studies may result in overestimation or underestimation of the true asymptomatic fraction [41, 42, 193].

Issues related to determining the true fraction of ASI stem from multiple factors. First, many studies reporting on ASI were cross-sectional surveys, often with convenience sampling and different testing eligibility criteria and settings, and were not designed to estimate the prevalence of ASI. Therefore, they are prone to significant selection biases. Second, the paucity of adequate follow-up hampers distinguishing between presymptomatic and asymptomatic individuals in many of these studies [41]. It is crucial to account for the development of symptoms not only at the time of virological testing since it is well established that symptoms can occur days after testing positive [43, 44, 194]. Based on the incubation period of SARS-CoV-2 [118, 166], a follow-up of 14 days from the last possible exposure to an index case (or first positive test if exposure is unknown) is recommended to exclude most presymptomatic cases [41]. Also, if the timing of SARS-CoV-2 exposure is unknown, assessment of prior symptoms is recommended to identify postsymptomatic cases, given the potential for long-lasting positivity of quantitative reverse transcriptase-polymerase chain reaction (qRT-PCR) testing in upper respiratory tract specimens following symptom onset (for weeks or even months) [43, 44, 194–197]. For example, it was reported that 43% of residents in countrywide screening in Iceland [198], 76% of pregnant women in a labor and delivery ward [199], and 81% of passengers and crew in an Antarctic-bound cruise ship [200] were asymptomatic. Due to the cross-sectional nature of these studies, it is not clear what proportion of these individuals were presymptomatic or postsymptomatic. In contrast, a study in a skilled nursing facility reported 56% of residents initially asymptomatic at the time of SARS-CoV-2 testing, of whom 89% went on to develop symptoms within one week, resulting in only 6% as truly asymptomatic [201]. Similarly, in a SARS-CoV-2 outbreak at a refugee shelter, 80% of individuals were asymptomatic at the time of testing but only 12% were asymptomatic during the 14-day follow-up period [202].

Third, some studies reporting a high prevalence of ASI only evaluated a narrow range of symptoms [41], leading to information biases. This usually happened in early 2020 when smell and taste disturbances and gastrointestinal symptoms were not widely documented. Not only are symptoms subjective and variably ascertained by screening questionnaires or self-reported symptom tracking, but patients may also be unaware of atypical, mild, and prodromal symptoms, may not recall symptoms upon retrospective assessment (recall bias), or may recount symptoms caused by other conditions. For instance, a high prevalence of ASI (88%) was reported in a homeless shelter, but occupants were asked only about the presence of cough or shortness of breath, with optional reporting of other symptoms [203]. Similarly, the initial report from Vo’, Italy noted that 43% of positive cases were asymptomatic individuals; however, symptomatic individuals were narrowly categorized as those with fever, cough, or at least two minor symptoms among a predefined list [204]. Both an inadequate follow-up and information biases in estimating exposure and symptom onset times lead to misclassification of some presymptomatic, paucisymptomatic, and postsymptomatic individuals as asymptomatic, likely resulting in an overestimation of the ASI prevalence.

Fourth, ASI estimates from serosurveys with uncertain timing of suspected exposure and antibody testing, and coupled with insufficient retrospective symptom assessment deserve caution given concerns with recall bias and the duration of detectable antibodies [41, 205]. Recall bias in serological studies may occur due to interviews or questionnaires gathering symptom information during a prior period, which might be particularly problematic with long or unspecified time windows. Antibodies are detectable in most individuals two to four weeks following symptom onset [206–208], hence positive IgG titers are out of the presymptomatic period and seropositivity results exclude recent infection. ASI percentages from serological studies have been variably reported. For example, serological studies have estimated an ASI fraction of 32% in England [209], 33% in Spain [167], 44% in hospital staff from Michigan, USA [205, 210], and 90% in Argentina [211]. Although two nationwide serosurveys on antibody testing [167, 209] were designed to achieve representative samples of community-dwelling individuals [212], their accuracy heavily relies on measurement-related factors (e.g., timing of testing, antibody test performance, retrospective symptom ascertainment), as discussed elsewhere [41]. Unlike serological tests, SARS-CoV-2 nucleic acid assays detect viral RNA and are useful for virological diagnosis and modeling transmission potential. Nevertheless, when better understood [213] and planned, seroprevalence studies may assist in identifying previously unrecognized infections and, alongside virological tests, allow more accurate estimates of the population-wide ASI prevalence rather than of the qRT-PCR-positive population [214]. Also, serial serological testing may help define antibody decay trends, which is useful to estimate ASI proportion in serological studies [41].

Fifth, confusing methodological definitions, different settings, and language barriers during international clinical assessment affect the generalizability of ASI estimates. Greater care and standardization with case definitions is justified to avoid misinterpretation of research findings, as occurred when a high rate of “undocumented infection” (86%)—apparently an admixture of ASIs, unreported symptomatic infections, and undiagnosed mild infections—was reported to be the source of 79% of documented cases [215]. This was misconstrued across scientific papers and social networks as ASI being responsible for the majority of SARS-CoV-2 infections [216]. Another unconventional and unnecessary term is “covert infection” [217], which was used in place of ASI in a systematic review [218]. Further, in a modeling study, researchers used the term “silent infections” to merge presymptomatic and asymptomatic infections [219]. Lastly, studies testing at a single time point or disregarding the time-changing sensitivity of qRT-PCR assays will rule out individuals with initial false-negative qRT-PCR results [220–222], thereby likely underestimating the ASI prevalence.

Of note, a well-defined cohort study of 47 SARS-CoV-2-infected individuals among 195 household contacts reported an ASI percentage at the time of testing of 17% (8/47) [185]. Five of the eight infected individuals were qRT-PCR negative at enrollment but positive during follow-up testing. Upon repeat qRT-PCR testing, ambispective granular symptom assessment, and 14-day follow-up of all participants, 13% (6/47) were classified as presymptomatic and 4% (2/47) were classified as postsymptomatic by date of sample collection, indicating that no individuals proceeded asymptomatically.

Several systematic reviews and meta-analyses addressing the conundrum of ASI have provided summary prevalence estimates from 16% to 25% [42, 214, 223–226]. Given the inclusion criteria used (clinical follow-up, quality of included studies, case definitions), these systematic reviews are more reliable and accurate figures than those from highly publicized narrative reviews [186] and opinion pieces [187, 188, 190–192]. Despite the scientific rigor of the articles cited above, generalizability is unclear and the wide prediction intervals of their pooled estimates reflect the considerable methodological and clinical heterogeneity among the studies included. Other systematic reviews with problematic inclusion criteria and definitions published much lower or higher estimates (e.g., 8% [218], 31% [227], 39% [228]) or did not meta-analyze data yet concluded that the proportion of ASI was “at least one third” [212] or was “not negligible in any population” [229].

#### The proportion of symptomatic SARS-CoV-2 individuals (in a broad sense)

Since all SARS-CoV-2-infected individuals are initially symptomless, the proportion of those with presymptomatic, symptomatic, postsymptomatic infection (the same individuals) can be indirectly estimated in the range of 75%–84% by subtracting higher-quality ASI proportions reported in available systematic reviews from the totality of infections [42, 214, 223–226].

Pooled estimates of the proportion of presymptomatic SARS-CoV-2 infection published in three systematic reviews are dissimilar (8% [218], 15% [228], 49% [223]). This raises several concerns. While pooled ASI proportions may be valid and useful when a systematic review meta-analyzes high-quality evidence, the case of presymptomatic infection is a different one. Meta-analyzing proportions of the stages of infection of the symptomatic individuals makes little sense not only because of the variable testing times, definitions, and follow-up in individual studies, but also because the presence of symptoms is not a fixed feature of infection. The pooled proportion of presymptomatic infection of an individual study usually reflects the specific moment of testing or study assessment (i.e., PCR testing) rather than exposure. As a result, the pooled proportion of presymptomatic infection might, at best, give an idea about how often infected individuals that will develop symptoms are symptomless by the date of testing across heterogeneous studies. Therefore, systematic reviews should instead analyze the methodological aspects of original studies and epidemiological parameters and timelines that influence both clinical presentation and transmission. Aggregate analyses of timelines detailing key events (e.g., exposure, symptom onset, changes in NPIs, contacts) and serial virological data are valuable to estimate infectiousness and transmission risk.

#### Differential transmission of symptomatic, presymptomatic, and asymptomatic infection

From a public health and clinical standpoint, the relevance of using the term “presymptomatic” in addition to “symptomatic” and “asymptomatic” lies in differential transmissibility features between infected individuals depending on symptom status and stage of infection. These features include secondary attack rates^17^ (higher for symptomatic and presymptomatic individuals) [42, 45, 224, 230–232], viral RNA shedding dynamics (longer viral RNA shedding and occasionally higher viral loads in symptomatic and presymptomatic individuals) [43, 44, 233], and modeling estimates of the contribution to transmission (higher proportions of SARS-CoV-2 infections are estimated to originate from presymptomatic and symptomatic individuals) [219, 234–238]. While these findings support a higher transmission risk for symptomatic and presymptomatic individuals compared with asymptomatic individuals, the latter cannot be dismissed as inconsequential to SARS-CoV-2 transmission [239, 240]. Symptom-based strategies (e.g., case detection and isolation, self-isolation) are necessary but insufficient given the difficulties in recognizing the onset of mild or atypical symptoms in addition to the risk of symptomless transmission. While vaccination rates progressively increase worldwide, multipronged preventive measures that do not depend on identifying symptoms (e.g., physical distancing, mask wearing, ventilation, hand hygiene) continue to be essential for controlling SARS-CoV-2 spread.

#### Accurate messaging and further research

Misclassification of infected individuals continues to cloud our understanding of COVID-19 and may impact policies to control SARS-CoV-2 transmission. In acknowledging the definitions reviewed in this section and the existing evidence on the proportions of infected individuals and their differential transmission risk, some claims in scientific articles and opinion pieces are misleading (e.g., “Most coronavirus cases are spread by people without symptoms,” “Asymptomatic persons are playing a major role in the transmission of SARS-CoV-2”) [241, 242]. Examples of more accurate and informative statements are “Most individuals with SARS-CoV-2 infection experience symptoms during the course of infection,” “About one in five infected people are completely asymptomatic,” and “SARS-CoV-2 cases are substantially spread by infected people both when they have symptoms and when they do not.”

Further research that incorporates nuanced definitions and systematic methods will enable a wider understanding of factors potentially influencing SARS-CoV-2 transmission such as viral load and the presence and onset of symptoms. Despite important advances toward understanding SARS-CoV-2 transmission dynamics, estimating the contribution of transmission is tricky and specific scenarios of transmission are extremely complex. Many aspects remain uncertain including the dual role of social behavior and biological features on transmission, evidence of presymptomatic viral load peak from empiric studies, and viral RNA shedding dynamics and infectious timeline of individuals with ASI. New studies will have to conduct rigorous analyses considering the influence of increasing vaccination rates on the clinical presentation of COVID-19. Also, there is a need for carefully designed studies that document persistent symptoms after acute illness, help understand COVID-19 aftermath, and improve care interventions, quality of life, and return to usual health of COVID-19 survivors with lingering symptoms.

### False dichotomy 4: Droplet vs. aerosol transmission of SARS-CoV-2

#### The long-standing dichotomy of droplets and aerosols

The COVID-19 pandemic has reawakened the long-standing dichotomy of respiratory droplets and aerosols in terms of their size and transmission distance [47, 243]. Droplets and aerosols are erroneously seen as categorical transmission modes instead of a continuum of respiratory particles influenced by particle size and density, emission composition, turbulence and direction of the exhaled jet plume, and interacting environmental conditions [48, 244]. Larger droplets (traditionally defined as >5–10 μm in diameter) stay aloft for shorter periods of time relative to their size, settle on the ground within seconds to minutes because of gravitational force, and are transmitted over short distances (usually < 6 ft or 2 m), although airflow can propel them farther across a room. Small-particle aerosols or droplet nuclei (traditionally defined as <5 μm) generally evaporate and disperse faster than they fall, remain in the air for minutes to hours, and travel longer distances. This outdated distinction between droplets and aerosols has been revised by aerosol scientists arguing that the correct size threshold to differentiate these particles should be 100–200 μm [245–247]. “Aerosols,” a term commonly used as a shorthand for “aerosol particles,” are defined as a stable suspension of solid and/or liquid particles in air smaller than the above size cutoff, whereas droplets are defined as liquid particles larger than aerosols [247].

SARS-CoV-2 transmission cannot be separated into the earlier dichotomy of stationary droplets vs. suspended aerosols or the newer dichotomy airborne vs. non-airborne. Transmission patterns are on a continuum rather than dichotomous [48]. Although several issues need clarification and discussion to achieve scientific understanding and effective public communication, no debate exists as to whether respiratory particles of varying sizes can be generated from an individual. Both aerosol-generating behaviors (e.g., coughing, sneezing, speaking, singing, shouting, breathing) [248–250] and medical aerosol-generating procedures (AGPs)^18^ [253] lead to the production of respiratory particles spanning a wide spectrum of sizes. To avoid dichotomization and better describe the behavior of respiratory particles, some researchers have referred to the continuum of aerosols and droplets of all sizes as a multiphase turbulent gas cloud (“puff”) of exhaled air [244].

#### The modes of transmission of SARS-CoV-2

Transmission of SARS-CoV-2 may occur via several biologically plausible routes and depends on multiple factors, including the infectious dose (or inoculum), virus viability, exposure distance and duration, environmental factors (temperature, humidity, precipitation, pH, airflow/ventilation, solar ultraviolet radiation, chemicals), and host factors (breathing rate, respiratory tract morphology, target tissues, receptor distribution, host barriers and immune responses) [49, 254–256]. Transmission risk in specific settings is further influenced by existing infection prevention and control (IPC) practices and public health interventions [257, 258].

As acknowledged by the CDC, SARS-CoV-2 transmission occurs through three non-exclusive modes of exposure to infectious respiratory fluids: 1) inhalation of infectious small fine droplets and aerosol particles, 2) deposition of these particles onto mucous membranes (nose, mouth, or eyes), and 3) by touching mucous membranes with hands contaminated by respiratory fluids or indirectly by touching inanimate surfaces with virus on them [50]. As transmission of infectious agents is complex and dependent on several factors, awareness of such distinctions is important for NPIs and public communication. Although the relative contribution of all transmission modes remains unquantified [49], substantial evidence exists in support of specific transmission modes. Close-contact respiratory transmission, via short-range (inhalable) aerosols and droplets, is the primary mode of SARS-CoV-2 transmission [48, 49]. Direct contact (physical) transmission and indirect contact transmission (or fomite transmission) play a minor role in propagating SARS-CoV-2 [46, 51, 155, 259]. Long-range aerosol transmission (traditionally known as airborne transmission) occurs situationally, under certain conditions such as prolonged exposure in enclosed spaces with inadequate ventilation [47, 50]. SARS-CoV-2 infections through inhalation at distances greater than 6 ft are less likely to occur than at close distances. The CDC has also emphasized that transmission due to inhalation and mucosal deposition of virus is effectively mitigated by existing intervention recommendations [50], such as well-fitted masks, adequate ventilation, physical distancing, and avoidance of crowded indoor spaces. Other transmission routes (e.g., conjunctival, vertical, fecal-oral, zoonotic), though possible or suggested, are regarded as insignificant based on existing evidence [46].

Airborne transmission—taken in its traditional definition of long-distance and respirable aerosols—is not the dominant or exclusive route for SARS-CoV-2 transmission [48, 49]. Conflicting and polarizing messages pertaining to SARS-CoV-2 transmission modes jeopardize pandemic response plans, resulting in public unwillingness to adhere to risk reduction practices. Exaggerating the frequency of a transmission route [260] prioritizes unnecessary IPC measures and social behaviors in hospital and community settings at the expense of effective interventions in place and undercuts public trust. Infectious disease transmission has important implications for deploying cost-effective IPC protocols and allocating resources to achieve the largest impact possible. Overstated evidence can lead to harmful policies. By amplifying findings from studies with methodological concerns and limited transferability of results [261, 262], some academics and laypeople have advocated the use of filtering facepiece respirators (FFRs) in routine healthcare or even in community scenarios [263–266], despite evidence showing that FFRs may not be necessary in some settings to reduce transmission risk [267]. This has led to risk perception disparities and public confusion.

#### Epidemiological evidence

Epidemiological data (outbreak, cohort, and case-control studies) help determine SARS-CoV-2 transmission mechanisms in real-world conditions. Theoretical modeling, laboratory-based, and *in silico* studies are useful as complementary sources of knowledge but are not necessarily reflective of the frequency of a transmission mode and the real-life situations, especially if they do not consider SARS-CoV-2 infectivity or are simulated in vastly different scenarios.

Several arguments support transmission through close contact with the infectious source [48, 50, 52]. First, the basic reproduction number^19^ (R_0_, 2–3) [268, 269] and household secondary attack rates (generally 10%–20%) [230–232] for SARS-CoV-2 are compatible with predominant close contact transmission rather than long-range aerosol transmission [47, 270]. Second, several observational reports of COVID-19 hospital cases and outbreaks have indicated that transmission-based precautions (TBPs) for routine care of patients generally work if instituted timely and consistently [48, 257, 271–284]. Hospital-acquired SARS-CoV-2 is rare in healthcare settings with robust IPC programs. The findings of some studies [285, 286] reporting an increased risk for SARS-CoV-2 infection among healthcare workers (HCWs), even when wearing adequate PPE, compared to non-HCWs do not immediately translate into predominant long-range aerosol transmission, especially when there is little or no consideration of the variation in IPC practices and PPE types [48], definitions of compliance and consistent wearing, AGP care exposure, breakroom or changing room exposure [48, 287–289], and community SARS-CoV-2 exposure of HCWs [290]. Medical masks have been demonstrated to reduce infectious titers of other respiratory viruses with similar transmission patterns [291]. Meta-analyses of clinical studies comparing medical masks with FFRs have reported no statistically significant difference in preventing respiratory viral infections (including those caused by seasonal/endemic coronaviruses and influenza) in HCWs [292–297]. The problem is that the evidence is heterogeneous and hindered by suboptimal PPE adherence and underpowered study designs. The need for higher-rated PPE should be calibrated to the degree of risk [298]. As many HCWs in clinical care (and potentially other essential workers) are at the highest risk for exposure due to proximity, duration, and infectiousness of patients [267], access to fit-tested FFRs is indicated for their safety. Medical masks reduce but do not eliminate aerosol exposure and therefore may offer incomplete protection for frontline HCWs and other HCWs that engage in near-range, face-to-face, sustained encounters with patients with known or suspected COVID-19, untested individuals, and/or individuals that are unable to wear masks [298, 299]. The value of FFRs outside of these circumstances is likely marginal but more research is needed [298]. Third, community-based reports generally support the effectiveness of the existing TBPs (if consistently and adequately instituted) [300–309]. Accordingly, both the World Health Organization (WHO) and the CDC have reiterated that current recommendations are in general effective against both inhalation and mucosal deposition of respiratory particles [50, 52].

Several SARS-CoV-2 outbreak studies have been published in different settings, including restaurants [310, 311], call centers [312], choir rehearsals [313, 314], indoor fitness and sports facilities [315–319], long-term care facilities [201, 320–324], correctional facilities [325], malls [326], churches [327, 328], flights [304, 329], social gatherings [330, 331], camps [144], ships [200, 303, 332], bus transportation [333], and acute care hospital settings [299, 334]. Many of these outbreak studies have been often cited by other reviews as evidence of airborne transmission. However, long-range aerosol transmission is a plausible explanation in only some of these settings [48]. Other modes of transmission cannot be ruled out and may fit the particular transmission conditions. In general, published clusters associated with long-range aerosol transmission are singular events with preventable circumstances, such as prolonged duration of exposure, lapses in the use of PPE, increased exhalation, indoor settings, and poor ventilation.

#### Laboratory studies and modeling data

Different types of laboratory studies have been conducted in an attempt to elucidate SARS-CoV-2 transmission. Some laboratory studies (e.g., using a 3-jet Collison nebulizer) have shown that experimentally-generated SARS-CoV-2 aerosols may remain infectious for up to 3–16 hours [335, 336]. Unfortunately, such studies under controlled laboratory conditions do not reflect physiological host processes and real-world environmental conditions related to viral transmission [270, 337]. Respiratory particle transmission and viability over long distances are subject to changes in ambient temperature, relative humidity, airflow/ventilation, solar ultraviolet radiation (sunlight), and chemicals leading to evaporation, supersaturation, dilution, or inactivation [49, 254–256]. Aerosol transmission, direct contact transmission, and fomite transmission have been experimentally demonstrated in multiple animal models [49, 338–343]. Furthermore, studies in non-human primates, and confirmed in humans, demonstrate that infected individuals exhale infectious aerosols, but this is highly variable across individuals and activities [344, 345].

Experimental, computational fluid dynamics simulation, and mathematical/numerical modeling studies have found that respiratory particles floating in the air can reach distances of 20–26 ft (6–8 m) or thereabouts [244, 265, 346, 347]. However, this does not mean predominant long-range aerosol transmission of infectious viral particles. While respiratory particles have a great capacity to travel long distances or linger in the air for some time, transmission risk hinges greatly on how much infectious virus those particles contain and the conditions of the environment. These particles will diffuse and dilute in the surrounding air leading to progressively lower virus concentrations.

Droplet dispersion experiments (e.g., using laser light scattering) have shown that aerosols can travel for long distances [265, 348–350]. However, these studies did not quantify infectious SARS-CoV-2 concentrations, which are likely substantially lower over long distances and under dynamic environmental conditions. Findings from Stadnytskyi et al. [349] relied on the independent action hypothesis, which states that each virion has an equal, nonzero probability of causing an infection (i.e., even a single virion can establish infection). This hypothesis remains scarcely tested and is unknown to be valid for humans and their infecting viruses including SARS-CoV-2 [270, 349].

Many studies have looked for evidence of viral RNA in ambient air samples and ventilation systems of hospitals [351–385]. Some of these studies detected SARS-CoV-2 RNA in some air samples [351–374], but other studies did not [375–384]. Several of the qRT-PCR-positive studies were not successful in isolating viable SARS-CoV-2 [351, 354, 357, 364, 366, 367, 371, 372], while others did not attempt to culture SARS-CoV-2 [355, 356, 358–363, 365, 368–370, 373, 374]. Two hospital-based studies have reported infectious SARS-CoV-2 in ambient air. The study by Santarpia et al. collected aerosol samples around six patients admitted into medical wards, characterized the size distribution of aerosol particles, and assessed the presence of infectious virus in different particle size ranges in the patient environment [352]. The authors demonstrated the presence of SARS-CoV-2 RNA and increases in viral RNA during cell culture of the virus from recovered aerosol samples, especially in particles with size < 1 μm. In another study, Lednicky et al. used an air sampling technology based on water vapor condensation to determine the presence of viable SARS-CoV-2 in hospital room air of two COVID-19 patients [353]. Viable SARS-CoV-2 was isolated from air samples collected 2 to 4.8 m away from the patients, with estimates ranging from 6 to 74 median tissue culture infectious dose (TCID_50_) per L of air. It is yet unclear the extent to which these findings represent an unmitigated risk in healthcare settings where PPE and other TBPs are properly applied. Identification of SARS-CoV-2 RNA and viable SARS-CoV-2 in air samples from healthcare settings lend credence for aerosol transmission in these settings but does not provide straightforward information on its frequency as a transmission mode for SARS-CoV-2. Nor is a hospital setting, with robust ventilation, air filtration, and PPE, comparable to risk or frequency in the community [257]. This similarly applies to fomite transmission, which is not considered a major transmission mode despite numerous laboratory-based studies conducting environmental sampling and reporting SARS-CoV-2 surface contamination and stability [386]. Nuance is needed when examining the evidence of air sampling studies instead of calling the retrieval of infectious SARS-CoV-2 a “smoking gun” [387].

Some studies conducting community-based SARS-CoV-2 RNA detection in air samples have reported negative findings, including those from cruise ship cabins [388], quarantined households [389], residential areas [354, 370], open public areas [354, 368], and transportation [368, 390]. In contrast, other studies have reported positive qRT-PCR-positive air samples from a variety of indoor or crowded public spaces [370, 391] and transportation [391, 392], with SARS-CoV-2 viability not assessed. Three additional studies assessing the presence of SARS-CoV-2 RNA in outdoor particulate matter (PM) in Italy and Spain found all air samples negative [393–395]. A modeling study estimated a very low average outdoor concentration of SARS-CoV-2 RNA (<1 RNA copy/m^3^) in uncrowded outdoor public areas in Italy, even in the worst-case scenario [396]. Conversely, researchers of one Italian study found that 20 out of 34 PM_10_ (PM with diameter < 10 μm) samples were qRT-PCR-positive [397]; however, concentrations of virus-laden particles were not examined and culture data were not provided. Although the implications of atmospheric pollutants on transmission remain elusive [53, 398], several studies (mostly ecological) and commentaries arguing about an association between air pollution and SARS-CoV-2 airborne transmission and mortality [347, 399–404] have sparked concern about PM acting as a carrier of SARS-CoV-2 and diffusing the virus in open environments. An ecological study about PM in several Italian provinces found a positive correlation between daily PM_10_ exceedances and COVID-19 cases [403]. The authors of this study hypothesized that the growth and severity of cases in Milan could be attributed to airborne diffusion and a “boost effect on the viral infectivity corresponding to the peaks of PM.” They also illustrated the “airborne route of transmission as a ‘highway’ enhancing viral transmission over 8 m.” No scientific evidence suggests or supports such claims. Available air pollution studies point to correlation rather than causation (i.e., highly polluted areas in some countries are characterized by large populations and increased rates of human interaction, and lockdowns reduce both air pollution and SARS-CoV-2 spread) [53, 398]. Furthermore, upon theoretical examination, the probability that atmospheric pre-existing PM scavenges virus aerosols is low [396]. Monitoring of SARS-CoV-2 RNA in outdoor PM is therefore unlikely to be an early suitable indicator of viral diffusion or pandemic recurrence [393, 394]. Some scientists have also speculated that airborne pollen [405] and sea spray [131, 132] may act as a modulating factor of SARS-2 infection and transmission, with only ecological data supporting an association for the former [406]. However, there is enormous potential for confounding due to several factors implicated in transmission of respiratory viruses, including well-known environmental factors such as ambient temperature. In addition, no evidence supports that pollen grains are carriers of SARS-CoV-2, much less does it provide information on their frequency and risk of transmission. A study of air samples collected in Germany and experiments to examine potential complexes between purified pollen of various taxa and SARS-CoV-2 reported negative findings—in terms of both viral RNA and virus-induced cytopathic effects [407]. While environmental exposome deserves further examination, evidence must be accurately communicated to avoid panic and misunderstandings.

In summary, a low level of air contamination has been demonstrated in both healthcare and non-healthcare settings thus far. The findings of the air sampling studies are related to the sampling methods and duration, storage and transferring conditions, the environmental setting, low viral concentrations, dilution effects, and ongoing IPC measures [408, 409]. Further, pressing issues concerning virological testing warrant discussion. qRT-PCR cycle threshold (Ct) values have been increasingly used as informative proxies for probable infectivity [196, 197, 410, 411]. However, viral nucleic acid detection by qRT-PCR-based assays does not equate to shedding of infectious, viable, culturable, or replication-competent virions [412, 413]. Viral load and Ct values have limitations [222, 414, 415]; their correlation depends on the gene targets used, the nucleic acid extraction system, among other factors. Detectable viral RNA exceeds infectious viral clearance [43, 44, 194–197] likely because genomic and subgenomic RNA persists as residual viral fragments or is protected by cellular membranes, and degrades slowly after the immune system has neutralized or lysed virions [412, 416]. Demonstrating virus amplification or cytopathic effect in cell culture, or virus quantification by plaque assays or TCID_50_ endpoint dilution assays are needed to infer viral replication and infectious virus [417]. Therefore, these are better surrogates for assessing transmission competency, although the sensitivity of viral culture may be a concern as well [222]. Unfortunately, infectious titer assays must be conducted in biosafety level 3 (BSL-3) containment, so routine measurement of infectious SARS-CoV-2 in clinical settings cannot be done. Further methods to quantify infectiousness [415] and reproducible research with emerging technologies to sample air particles are needed.

#### Unknowns in SARS-CoV-2 transmission

There are virological and aerobiological unknowns of SARS-CoV-2 that are germane to elucidating transmission modes, including the minimum infectious dose, the size of particles with major relevance for transmission, and virus concentrations and viability in respiratory particles. In addition, several factors that influence transmission warrant study: particle emission and composition, particle size transformation and distribution over time, and environmental parameters (e.g., temperature, humidity, indoor/outdoor setting). High-quality research is needed to better understand these aspects and attempt to estimate the relative contribution and importance of the transmission routes of SARS-CoV-2. However, this is challenging because of the complexities in transmission [49], including the fact that respiratory particles containing infectious SARS-CoV-2 are highly variable in different individuals and with different activities [344, 345].

#### The use of the term “airborne,” the lack of nuance, and inaccurate analogies

It has become clear that aerosol transmission is an important transmission mode. However, there is controversy about using the term “airborne” due to varied existing definitions, meanings, and implications [418], including the ordinary meaning of the word (carried in the air) and scientific conventions and specialized meanings referring to long-distance aerosol-based transmission.

While some scientists advocate the use of the term “airborne” as a simple term to use in risk communication with the public, the plain usage of this word when referring to SARS-CoV-2 transmission is technically reductionist and ambiguous. The flagrant use of the term “airborne” without providing nuance can be misinterpreted. For example, if the public wrongly believes that transmission occurs overwhelmingly from aerosols over an extended distance and time, they may reject guidance to wear medical masks or cloth face coverings (given their limited aerosol filtering efficiency in comparison with other facepieces), hoard FFRs, or feel that distancing precautions are futile. Likewise, if the public believes that the virus spreads extensively in the outdoor air and travels down blocks or across buildings, this may lead to potentially dangerous practices such as closing all windows in residential areas.

From a public health standpoint, the term “airborne” is not actionable on its own because it offers no clear guidance on how to curtail exposure risk. Simplistic messages and press article headlines, such as “The coronavirus is airborne,” "It is in the air,” and “Coastal breezes likely carry coronavirus” [131, 132, 419–422] require nuance to provide effective and accurate risk communication in public health and to avoid misunderstandings of viral transmission and airborne fearmongering. This has been exacerbated by scientific commentaries claiming with selective citations that airborne transmission is the predominant mode of SARS-CoV-2 transmission, without addressing terminology, practical implications, and critical aspects in public health risk communication and community engagement [260, 423]. Miscommunication of transmission modes precludes harm reduction approaches (e.g., enjoying outdoor spaces such as beaches [132], and avoiding indoor gatherings) by failing to acknowledge that outdoor airborne transmission is low, particularly if the setting is uncrowded [40, 138, 139].

Inaccurate analogies have also been increasingly used. Cigarette smoke has been mentioned as a proxy for SARS-CoV-2 infection risk [216]. While this may meet the physical properties for aerosol scientists, analogies that intertwine sensory reception, such as smelling volatile organic compounds in smoke, can be misleading in terms of respiratory protection efficacy. The possibility to smell a vapor while wearing a fitted N95 FFR (or equivalent PPE) can mislead HCWs into thinking that their PPE is not effective.

#### Toward a multidisciplinary agreement on actionable terminology

Given the societal challenges of COVID-19, never has there been greater need for meaningful interdisciplinary dialogue. Agreement on actionable terminology that respects different fields is long overdue. The pandemic has underscored the continuum and spectrum that is viral transmission. Such complexities should be addressed with collaborative efforts to communicate in a way that meets the needs of all parties. Nuance and complexity can be understood by the public if communicated clearly and transparently. Public health messaging and risk communication should mention that respiratory pathogens may transmit over long distances via the air under specific conditions, while making clear recommendations about effective mitigation measures. Central to the use of accurate terminology is the risk assessment of indoor vs. outdoor spaces and banishing the thinking of viral transmission as miasma or an insidious trail containing endless infectious virions.

Rather than droplet vs. aerosol or airborne vs. non-airborne dichotomies, evolving terminology and science communication for respiratory pathogens should move toward reflecting the nuance of transmission and effective interventions [48]. Broadening the “airborne” definition to inhalable aerosol/droplet exposure or respiratory transmission allows new avenues to be explored and reconciles seemingly contradictory data and disciplines. Furthermore, discussing enhanced respiratory precautions and differences between long- and short-range, as well as risk in terms of types of exposure and activities can effectively inform subsequent public health interventions. As long-range aerosol transmission is situational, these circumstances can be explained through an increase in risk factors as dimmers rather than on/off switches. Both the WHO and the CDC have utilized this approach with communicating risk, with an emphasis on proximity, activity, environment, ventilation, NPIs, and vaccination status [32, 50, 52, 55, 424].

Bridging the interdisciplinary communication barriers and disagreements between the medical and engineering fields has proven complicated. Although academic disagreements may be valid and should not be met with hostility, narratives of misinformation and false dichotomies cause harm or do little to address the global needs for COVID-19 mitigation. There have been large-scale, continued attacks on those working in public health, which undermines public trust and is counterproductive to the pandemic response. Different disciplines should work together [425], instead of taking an adversarial position against public health agencies like the WHO and the CDC [426, 427], which is decidedly not constructive.

In the end, the unresolved semantic dilemma warrants interdisciplinary efforts from the full range of experts, including medicine, epidemiology, occupational hygiene, engineering, and fluid physics, seeking a classification framework that recognizes both technical knowledge and practical implications in the context of public health and reconciles with real-life evidence without drawing inaccurate or unduly alarmist conclusions from available studies. Nuanced and transparent communication efforts, coming from those actively working to advance health and research amid the pandemic and facing the challenges of media representation of terminology, are valuable endeavors.

### False dichotomy 5: Masks for all vs. no masking

#### Culture war and the false dichotomy of community mask wearing

Preponderantly framed as a medical intervention in the past, face masks have become embedded as a social practice informed by expectations and norms amid the COVID-19 pandemic [56, 428]. Masks have provoked a culture war and vigorous debates in many regions, with a volte-face in attitudes from mocking mask wearers earlier in the pandemic to shaming mask abstainers later [19, 54, 429–431].

On one side of the politically charged false dilemma about community masking, some “pro-mask” academics and armchair epidemiologists have hyped masks with overconfident slogans (e.g., “Just wear a mask, it’s common sense,” “The science behind masks is simple and clear,” “Masks increase rate of asymptomatic cases”) [18, 57, 190], stigmatizing terminology to refer to people not wearing masks (e.g., “deviants”) [428], and inaccurate analogies with parachutes and other accessories [432–434]. Also, some modeling/simulation studies, quasi-experimental studies, and ecological studies [280, 435–446] were overinterpreted in social and mass media without due acknowledgment of their limitations, including confounding. With well-meaning but incendiary rhetoric [431], some mask proponents overstated the benefit of masks in preventing SARS-CoV-2 transmission and downplayed many considerations needed for community masking uptake and public trust. Likewise, existing evidence was misinterpreted to advocate further benefits of mask wearing related to reduced COVID-19 severity (or increased ASI rates), and protective immunity via reducing the viral inoculum (one of these papers was a preprint withdrawn by the authors) [190–192, 447–449].

On the other hand, there have been two “*anti-mask”* groups or counterpublics shaped by their hostile stance toward masking. One seems to ignore the need for and utility of complex systems methodologies, plausibility designs, and diverse evidence approaches [450–452] to study population-level interventions while staunchly upholding evidence-based medicine tenets (extended from biomedicine traditions and philosophies) and awaiting “definitive” randomized controlled trials (RCTs). The other has vociferously disparaged the use of “muzzles” or “face nappies” based on unwarranted or negligible physiological concerns (e.g., increased risk of hypercapnia, clinical worsening of infected individuals, increased risk of skin infections) [453–455], infringement on libertarian values [19, 456, 457], toxic masculinity [458, 459], or plain mask denialism [460, 461]. Unsurprisingly, deep-seated conspiracy theories, scientific illiteracy, strong political views, and counter-visualizations^20^ have stoked the anti-mask sentiment of the latter group, aiming to overturn mask recommendations and mandates [19, 462].

Setting up a binary choice between “masks for all” and no masking is overly simplistic. Further, reinforcing a view of “altruistic” vs. “selfish” people fosters a damaging binary [56]. Claims from eminent individuals polarized at either side of this false dichotomy (i.e., either “mask absolutists” or “mask abstainers”) have promoted a culture war. The public should be treated as stakeholders with legitimate input into mask debates, not just as adopters, resisters, or “deviants” that need to be persuaded or forced to wear masks [56].

#### The science of masks is not straightforward or simple

Masks—with their benefits and caveats [57]—are not a panacea or a hoax, nor are they mere symbols and commonsense interventions of the pandemic response [54]. There is merit in appraising different types of evidence on respiratory viruses and masks, particularly as this is the case of a complex public health intervention. Evidence on masks varies across study designs, settings, and populations; mask types and designs; mask-wearing purposes; and clinical and microbiological outcomes assessed. Medical masks and FFRs have been shown to prevent respiratory viral infections in healthcare settings [262, 293, 297, 463–466]. In general, clinical studies comparing medical masks (also known as surgical or procedure masks) with FFRs have reported no statistically significant difference in preventing respiratory viral infections in HCWs [292–297]. As for community scenarios, before COVID-19, there had been evidence with mixed results for medical masks used by healthy and sick people in households, university residences, schools, and mass gatherings (the Hajj pilgrimage) but much less research on cloth face coverings (also known as cloth or fabric masks) to prevent onward transmission (source control from an infected person) and contracting infection (personal protection of an uninfected wearer) [463, 467–469]. Researchers of the only existing RCT on cloth face coverings, carried out in 14 hospitals in Hanoi, Vietnam, initially cautioned against the use of cloth face coverings to protect against clinical respiratory illness, influenza-like illness, and laboratory-confirmed respiratory virus infection, compared with medical masks [470]. A post hoc analysis found that the risk of infection was doubled if cloth face coverings were self-washed by hand by the wearers rather than laundered in the hospital [471]. Face coverings laundered in the hospital were as protective as medical masks. The majority of existing healthcare and community studies have focused on medical masks and FFRs, and have examined clinical endpoints and influenza-related outcomes. Direct evidence of mask use related to infections caused by coronaviruses (not SARS-CoV-2) is relatively sparse [472]. Of note, the COVID-19 pandemic has prompted abundant research on SARS-CoV-2 and masks, which is discussed below.

Mask filters collect particles through a combination of mechanisms including inertial impaction, interception, diffusion, and electrostatic attraction [473]. Medical masks have higher and more variable particle penetration rates (~10%–70%) than N95 FFRs (or equivalent), which present low particle penetration rates (<5%) [474–478]. Several filtration studies of cloth face coverings have reported widely variable filtration efficiency and breathing resistance (breathability) estimates depending on the mask design and textile features (i.e., fabric microstructure, permeability, electrostatic properties, number of layers) [467, 479–491]. Among cloth face coverings, multilayer non-valved masks made of hybrid, closely-woven fabrics show the best filtration efficiency and overall acceptable wearing comfort [55, 58, 492–494]. Facial fit, an aspect critical to minimize both outward and inward leakage around the facepiece edges and to improve filtration performance, has been increasingly studied. Several techniques have been suggested (e.g., use of mask fitters, nose wires, nylon hosier sleeves, rubber bands, or hair clips; knotting and tucking the ear loops; cloth mask placed over another mask) [477, 478, 486, 495]. However, gaps in consistent communication with the public remain. Mechanistic evidence has demonstrated source control efficacy of medical masks in reducing influenza virus and human seasonal/endemic coronaviruses respiratory emissions from symptomatic individuals [291, 496, 497], as well as some protection against influenza virus afforded to the wearer [498]. Likewise, fluid dynamics simulation and experimental studies support the role of masks in limiting the spread of respiratory emissions [348, 499–502].

As for direct evidence on SARS-CoV-2, Ueki et al. conducted SARS-CoV-2 experiments with different facepieces and two mannequin heads facing each other to simulate source control and personal protection [503]. Medical masks and cloth face coverings were 57%–58% effective in protecting others and 37%–50% in protecting the wearer. N95 FFRs performed better with 86%–90% source control efficacy and 96%–99% personal protection efficacy. However, since variations in mask efficacy can be largely explained by the context of SARS-CoV-2 transmission (level of infection probability and virus abundance), medical masks and well-designed face coverings should be effective under virus-limited situations [267].

Several COVID-19 observational studies across diverse community scenarios [300–309] have suggested a benefit from masks in mitigating the transmission of SARS-CoV-2. On the other hand, there are the RCTs, which are presumed to provide the highest quality data. However, RCTs can hardly capture the complexities related to viral transmission and public health interventions [452]. Furthermore, large-scale mask RCTs related to SARS-CoV-2 are difficult to conduct given practical and ethical issues (e.g., involving no-mask controls raises an ethical dilemma regarding the principle of equipoise) and the existence of alternative types of evidence. Yet, two community-based RCTs have been conducted during the COVID-19 pandemic. One is the RCT DANMASK-19 that recruited 6,024 Danish citizens to evaluate the effect of medical masks recommendation in protecting against SARS-CoV-2 infection. This study found a non-statistically significant reduction in infection in the mask group vs. the non-mask group [504] (odds ratio 0.82, 95% confidence interval [0.54–1.23]). While medical use in this study led to a ~20% personal protection from incident SARS-CoV-2 infection, the study sample size was not enough to determine statistical significance. Because of methodological limitations of this study in addition to being underpowered (e.g., individual-level randomization, low mask adherence, serological diagnosis) [505, 506], the findings do not disprove the effectiveness of community masking. The results of this study, however, may reflect the personal protective effect (not source control) of a mask recommendation in Denmark at the time (when the community incidence of infection was modest). The other mask RCT is a yet unpublished study conducted in Guinea-Bissau [507]. This cluster-RCT (which thus allows the assessment of source control) will complete enrollment of around 40,000 participants by August 2021. Of note, this community-based study aims to assess the effect of wearing locally-sewed cloth face coverings on COVID-19 severity and mortality. This study’s outcome is clinical and not based on tests (personal communication). Although it may be able to provide some clarity on the science of cloth face coverings, this study raises ethical concerns. The choice to conduct an RCT with a control group not provided with masks more than a year into a pandemic where other types of evidence suggest their effectiveness deserves scrutiny. Furthermore, while the study protocol was designed with Danish and Bissau-Guinean researchers, conducting this trial in Africa rather than Europe or North America raises potential issues of medical racism and colonialism.

Whereas observational epidemiological studies are likely to overestimate masks' effects due to residual confounding, experimental epidemiological studies are likely to underestimate effect sizes due to both suboptimal adherence in the intervention group and contamination (mask wearing) in the control group [508]. Therefore, the real effect size is likely between the estimates seen in those two types of study, with the maximum benefit of masking potentially resulting from the combination of source control and personal protection. Also, laboratory experiments in animal models—with their inherent limitations—have provided evidence on the efficacy of masks in preventing SARS-CoV-2 transmission [340].

While efficacy (performance in controlled or ideal conditions) and effectiveness (performance in usual or real-world conditions) are not synonymous [450, 509], a large consensus and a growing body of literature have moved forward the uptake of community masking as part of comprehensive NPI bundles or “policy packages” aimed at preventing infections caused by respiratory viruses including SARS-CoV-2 [55, 58, 261, 262, 295, 464, 508, 510–516]. Importantly, a fact undergirding community mask wearing during the pandemic is the risk of transmission, not only from symptomatic individuals, but also from presymptomatic and asymptomatic individuals (discussed in section 3). All in all, the intricate evidence base on the efficacy and effectiveness of masks explains the confusing messaging by public health officials about masks throughout 2020 and why mask policies flipped as scientists gathered data [23, 31, 55, 58, 512].

Alternatives to medical masks and cloth face coverings have been sought. In the face of limited data, face shields or visors have been suggested to provide some advantages over face masks in terms of eye protection, frontward airflow protection, no hand-to-face contact, breathability, full-face visibility, reuse, and disinfection [261, 517–520]. However, variable design (shape, materials) of face shields and upward, downward, and sideways leakage jets from the edges, seams, and joints are major issues [482, 500, 521–523]. Face shields are therefore considered to provide a level of eye protection only [55, 424]. The performance of clear masks and modified face shields remains largely untested. Also, although masks and FFRs with exhalation valves may ease breathing, they are discouraged for source control since the valves bypass the filtration function for exhaled air by the wearer [55, 424].

#### Policymaking about masks and issues with compliance and mandates in the community

Many countries and regions with community-based transmission of SARS-CoV-2 have recommended or mandated the use of commercial or homemade cloth face coverings or medical masks to slow down the impact of viral spread [57]. This was particularly reasonable when population exposure increased as lockdowns ended. From a public health standpoint, mask advocacy has led to reflections over the way policies have been developed and communicated [54, 524], and over the ideological distinction between applied and academic epidemiology [23]. Some scientists and academics have invoked the precautionary principle^21^, not only to advocate changes in TBPs and to guide public health policies in general [263–265, 419, 529, 530], but also to argue the case for universal masking [432, 439, 531–533]. If the benefits of masks are to be considered (i.e., reduction of respiratory infectious disease transmission, mutual protection, positive prosocial signaling), potential downsides should not be utterly disregarded [55, 59]. The latter include shortage of medical masks and FFRs for HCWs [534, 535], cross-contamination due to inappropriate mask wearing [536, 537], risk compensation or complacency toward other preventive measures (evidence in favor [538–540], evidence against [541–547]), psychosocial effects (e.g., threats to autonomy, psychological relatedness, competence) [455, 548, 549], communication and learning difficulties [518, 550–555], physiological effects (e.g., subjective breathing discomfort or difficulties^22^, skin problems, headache, ocular dryness and irritation; these effects are more likely if there is a related predisposing condition) [454, 455, 556, 562–565], and environmental pollution from mask waste [566–569]. Of note, these lingering concerns are not reasons to refrain from community masking (using medical masks or face cloth coverings) but are opportunities to maximize the benefits of masking, improve mask designs, and sharpen public health policies and messaging. The benefits of wearing masks outweigh the potential harms¸ especially when there exists widespread community transmission of SARS-CoV-2.

In addition to three essential mask parameters (filtration, fit, breathability), proper and consistent wearing of masks influences their effectiveness [55, 57]. Training and guidance on correctness and consistency of mask usage are therefore crucial. Improper donning and doffing, usage of ill-fitting masks, and inconsistent mask usage point out challenges in scientific communication, health education, policy implementation, community outreach, and surveillance [57]. Mask adherence is contingent on aspects beyond mere “discipline”: knowledge about the virus, risk perception, social acceptability of masks, perceived efficacy, trust in government and health agencies, public engagement with science, health literacy, messaging from various sectors, past experiences with masking (e.g., for other respiratory virus epidemics or PM air pollution), mask comfort, consumer appeal, degree of enforcement by public authorities, accessibility (no supply issues), and affordability (no resource constraints) [4, 57, 59, 469, 570–572]. The psychological effects of masks are culturally framed and shape acceptance and adherence [54, 510]. Mask policies aimed at fostering uptake should reflect the complex and contested sociocultural meanings and implications of mask wearing [56, 428]. Studies examining sociocultural and psychological factors underlying public masking amid the COVID-19 pandemic are therefore vital to identifying motivators, barriers, and disparities, and formulating behavior change strategies that encourage and sustain appropriate mask wearing [469, 550, 572–581]. A study found that inducing empathy for people most vulnerable to SARS-CoV-2 promoted the motivation to adhere to physical distancing and mask wearing, whereas simply providing information about the importance of these measures did not [580]. A study exploring perceptions of face coverings via focus groups found that the most prevalent motivation was to protect or respect others, while barriers included discomfort, misinformation, and autonomy perceptions [574].

Another concern of masking is that of being compulsory and generalized. Haphazard compliance with mask recommendations amid a pandemic may justify mask laws. Behavioral experimental evidence showed that mask wearing signals prosocial concerns and may reflect a social contract where voluntary policies can trigger insufficient compliance, intensify stigmatization, and be perceived as less fair as opposed to mandatory policies [547]. However, issuance of blanket laws and punitive enforcement involves a trade-off with personal freedom. This might be counterproductive by further politicizing mask wearing, deepening structural inequalities, triggering active resistance and violence, and eroding public trust, particularly in regions with zero or little SARS-CoV-2 transmission [54, 582]. For the same reasons, mandating masks in circumstances that provide marginal benefit such as outdoor spaces is inconvenient. Therefore, mask mandates—targeting specific settings and situations—should only be issued upon careful analysis of the legal challenges and local implications. Governments enforcing population-level masking should ensure the availability of masks and develop plans for free provision of masks to populations that might experience barriers to access [31]. For instance, public service providers could be mandated to have a stock of masks and educational aids for users, and private businesses could offer masks to customers out of enlightened self-interest.

Duckworth and colleagues outlined three sensible steps during the transition toward acceptance of community mask wearing [583]: 1) “from effortful to easy,” 2) “from unclear to understood,” and 3) “from unconventional to expected.” Such an approach relies on education and effective public health communication. Permanent education campaigns and harm reduction-based approaches from both the government and the public are preferred over purely coercive approaches and patronizing exchanges (e.g., shaming, excessive fines, imprisonment, violence, criminalization) to attain the desired results regarding mask wearing and avert social divides [54, 57, 583, 584]. Lamentably, there are countless examples of the latter approaches, which are unlikely to foster masking and end the mask wars [54]. In public settings, penalties for not wearing a mask—if not limited to restricting access to a service—should not be excessive or unfair.

Any mask policy (and policy in general) must engage with the potential for inequality and social exclusion [56]. There is a need to address the impact of mask policies on vulnerable and marginalized groups, including D/deaf, hard-of-hearing, or visually-impaired people who substantially rely on lip reading, facial expressions, or unmuffled speech for communication [550, 551, 554, 555]; racial groups being asked to tip their masks, harassed for concealing their face, or disproportionately penalized for not wearing masks [548, 549]; and rural and poor populations without access to government information channels and online health warnings [74]. The absence of tailored policies risks these individuals becoming isolated, neglected, or stigmatized.

#### Smart masking, not universal masking, in the community

Publications that advocated universal masking for the public [432, 439, 442, 446, 531–533, 585, 586] omitted nuances regarding viral transmission dynamics, risk communication, and sustainability. Also, there was limited consideration of social sciences aspects, including how mask policies might play out in practice [54, 56].

Masks have become normalized during the COVID-19 pandemic, and therefore the quandary of yes/no has been replaced with a debate about who, where, when, how, and what type of mask should be worn [55]. Aligned with the WHO risk-based guidance on masks (first issued on 5 June 2020) [55], a smart masking approach seems more appropriate than universal masking in community settings. The term “universal” entails all persons, places, and times, but some exemptions for masking are legitimate and reasonable because of particular benefit-risk assessments [54, 587]. Mask exceptions should not be seen as symbolic rejections of the pandemic [54].

For instance, some individuals are unable or contraindicated to wear a mask (e.g., people with some breathing difficulties, intellectual disability, psychological distress, hearing loss) [518, 551, 555, 588], and masking of children may prove challenging [589–591]. Clear masks and face shields have been discussed in the literature as potential alternatives for these individuals. If face shields are worn in the context of mask non-availability or difficulties, the wearer should ensure proper design to cover the sides to reduce leakage [55, 424]. The benefits of wearing masks in children to prevent SARS-CoV-2 transmission should be weighed against potential harms associated with wearing masks, including social, communication, and developmental concerns, feasibility, and discomfort [591].

Furthermore, not all settings and activities allow mask wearing or confer the same risk of SARS-CoV-2 infection (discussed in section 2). The case for mask wearing is strongest in higher-risk scenarios such as crowded spaces, indoor venues, and unventilated places [55]. The case for mask wearing is weakest in marginal-risk scenarios such as outdoor and uncrowded environments where physical distancing and ventilation may be ensured (e.g., people engaging in outdoor activities, people driving alone). Additional exemptions from mask wearing include those scenarios where the mask would interfere with a particular activity or occupation (e.g., people eating, performers who require clear enunciation or being recorded, high-intensity or professional athletes). Since households may represent scenarios where routine appropriate masking is impractical for members, the case for mask wearing in households is strongest when non-household members are visiting or when a household member (who lives with other people) is infected or has been potentially exposed to SARS-CoV-2 because of a recent potential exposure (e.g., occupational exposure, crowded settings, travel) [31, 55]. Mask policies directed toward high-risk settings, and not toward low-risk activities, are expected to foster mask adherence and acceptance and decrease mask-related discomfort and fatigue [54, 59, 570].

#### Acknowledging uncertainty and countering misstatements

Some uncertainties still exist regarding the wearing of face masks and coverings as a measure to prevent or mitigate SARS-CoV-2 transmission. There are COVID-19 research opportunities to obtain direct and actionable evidence on the effectiveness of specific cloth face covering designs in community scenarios, extended use and reuse of cloth face coverings, the impact of diverse approaches to mask adoption, alternatives that are more comfortable and more environmentally friendly, downsides of masking, additive effectiveness of cloth face coverings and face shields in the community, and attitudes, beliefs, and behaviors toward masking in the long term [57, 59, 510, 517, 524].

The evidence around the relationship between mask wearing, SARS-CoV-2 inoculum, COVID-19 severity, and immunity has been poorly addressed and misrepresented in several viewpoint articles and scientific opinions [191, 192, 448, 449]. The hypothesis that mask wearing reduces COVID-19 severity, increases ASIs, and promotes immunity (“variolation”) has been challenged [60, 205, 592, 593]. As of writing, two comprehensive reviews by our group on the topic are undergoing peer-review and will be soon published (personal communication). Overstating the effectiveness of masks or the existence of benefits additional to curbing viral transmission may lead to false expectations and increased exposure to high-risk places, social gatherings, and leisure activities (in the absence of full vaccination), which in turn may end up undermining trust in pandemic response efforts when people, exhausted from the pandemic and the response, realize masks are not infallible, severe cases still occur, and the pandemic has not fizzled out.

A key aspect of mask advocacy is accurate messaging, which includes acknowledging the limited utility of mask wearing as a single intervention and cautioning against it as a sufficient alternative to a multilayered use of other NPIs, including physical distancing, ventilation, and limiting time in crowded spaces [55, 57, 424]. The main arguments should be based on scientific evidence rather than on moralistic stances and virtue signaling [54]. It is monumentally frustrating that academics both supporting masks and calling for well-crafted messages, nuanced (not universal) guidance, and further evidence have been misrepresented as anti-mask and accused of flagrant disregard for human lives by some universal masking advocates. The palpable sense of urgency in the COVID-19 pandemic requires a dispassionate discussion and weighing of benefits, risks, and uncertainties along with swift data-driven decision-making that accounts for the cases for and against public health interventions [8, 17, 524, 594, 595].

### False dichotomy 6: SARS-CoV-2 reinfection vs. no reinfection

In April 2020, the Korea Disease Control and Prevention Agency (KDCA) investigated 116 patients previously infected with SARS-CoV-2 who tested positive by qRT-PCR upon having met discharge criteria, including negative diagnostic tests [596, 597]. This sparked intense public concern in regard to whether these observations indicated scant or absent protection from SARS-CoV-2 reinfection—defined as the subsequent infection of a host with the same microorganism. Fears of continual cycles of reinfection owing to weak stimulation of adaptive immune responses followed this publication. It was also highly plausible that the positive samples were due to the performance limitations of qRT-PCR diagnostics. The debate around SARS-CoV-2 reinfection has two opposing views: 1) infection and recovery do not confer immunity, which results in the potential for cyclical reinfection; and 2) infection results in protective immunity that removes any possibility for reinfection. However, the situation is inherently more complicated. To address the SARS-CoV-2 reinfection conundrum, it is necessary to revisit how long and how well the immune responses are protective against SARS-CoV-2 [598], and differentiate reinfection, re-detection, and recrudescence.

#### Reinfection and natural immunity

The question of potential fading immunity and reinfection has been present since the beginning of the pandemic. Much of the concern arouse because infections by coronaviruses such as HCoV-NL63, HCoV-229E, HCoV-OC43, and HCoV-HKU1 generally confer short-lasting protective immunity (6 to 12 months) [599]. In addition, past investigations of severe acute respiratory syndrome (SARS) and Middle East respiratory syndrome (MERS) epidemics have suggested that IgG antibodies are detectable up to nearly two years and one year, respectively [600]. Early analysis of antibody responses from convalescent COVID-19 individuals suggested that neutralizing antibody levels were detected following symptom onset and remained elevated during the two-week follow-up period [601]. Also, a population study in Iceland showed that antibodies against SARS-CoV-2 did not decline within four months after diagnosis [602]. Reassuring data have accrued since, with several works supporting humoral immune responses to SARS-CoV-2 for at least 5–7 months [603, 604] and immunological memory (especially T-cell mediated) for at least 6–8 months [605]. Epidemiological analyses have reported natural immunity protection from reinfection for at least 6–12 months [61, 64, 604, 606, 607]. Protection could go beyond these estimates because of the complexity and robustness of immune responses, though it is also acknowledged that the induction and durability of immune responses—both humoral and cellular—are heterogenous across individuals and may be shorter in some [608].

As an uncommon feature of SARS-CoV-2, reinfections are expected when immunity wanes or pathogen’s antigenicity evolves leading to immune evasion. The first confirmed case of SARS-CoV-2 reinfection in the USA was a 25-year-old male patient who was reinfected nearly two months after his first positive test, with the two symptomatic infection episodes separated by two negative nucleic acid tests at different time points [609]. Although the patient tested positive for IgG and IgM antibodies upon reinfection, antibody testing was not conducted after the first infection. Next-generation viral genome sequencing showed that the sequence variability between the two virus isolates belonging to Nextstrain clade 20C was too great to be explained by evolution within the patient alone. Likewise, initial reports of SARS-CoV-2 reinfections confirmed by viral genome sequences were identified in other individuals from the USA [610, 611], Hong Kong [612], the Netherlands [613], South Korea [614], Belgium [615], Ecuador [616], India [617, 618], Qatar [619], Brazil [620–622], United Kingdom [623, 624], South Africa [625], and Colombia [626]. Additional cases from Brazil [627], Peru [628], and Colombia [629] were presumably SARS-CoV-2 reinfections, but no sequencing and phylogenetic analysis were conducted due to limited resources. The age of all these individuals (with confirmed reinfection) ranged from 21 to 92 years and the intervening period between first infection and reinfection for these cases ranged from 48 days to 9 months. Most individuals had no pre-existing known immunodeficiencies (except for a Dutch woman suffering from Waldenström macroglobulinemia [613]). Most individuals were symptomatic during both the first episode and the reinfection. Two individuals that were asymptomatic during the first infection remained as such thereafter [617]. Several individuals presented increased severity upon reinfection compared to first infection [609, 611, 613, 616, 622–625]. Cases in which the second episode was less severe raise the possibility of partial immune protection [62].

The limited diagnostic data available from the first wave of infections as well as the supportive evidence required to publish descriptions of reinfections^23^ has impacted our appreciation for the frequency of these events [62, 63]. More recently, large observational studies on reinfection have been published. A large, multicenter cohort study among HCWs in England reported an 84% lower risk of infection with a median protective effect observed seven months following primary infection [606]. This period was deemed the minimum probable since seroconversions not associated with a positive PCR test were excluded at baseline. A study in Italy found a 94% lower risk of reinfection among patients that had recovered from COVID-19 compared with patients with primary infection [61]. Natural immunity appeared to confer protection for at least a year. A matched cohort study nested in a representative sample of the general population in Switzerland found a 94% lower hazard of reinfection among SARS-CoV-2-seropositive participants, compared with seronegative controls, for at least eight months after initial serology [607]. Further, in a population-level observational study encompassing PCR-tested individuals in Denmark, researchers estimated that past SARS-CoV-2 infection conferred ~80% protection against reinfection, decreasing to 47% in individuals aged 65 years and older [64]. The overall estimate did not vary significantly by sex or over follow-up time (3–6 months vs. ≥7 months). In contrast, another large study of laboratory-confirmed cases in Qatar determined that the risk of reinfection was only 0.02% [619]. However, unlike this study, the Danish study involved a far greater proportion of asymptomatic individuals, who are likely to elicit relatively marginal immune protection [630]. Finally, the Alpha variant (clade 20I/501Y.V1 or lineage B.1.1.7) was not associated with an increased risk of reinfection in a study conducted in the UK [631], which is consistent with further evidence indicating minimal or no immune evasion associated with this genetic variant.

#### Re-detection

Although SARS-CoV-2 reinfection is possible, it is not responsible for the high number of post-discharge positives found amongst patient cohorts, and thus argues for diagnostic limitations as a major culprit. Persistent or intermittent RNA viral shedding leading to re-positive cases^24^ has been widely reported [632, 633]. For instance, a study assessed a group of recurrent-positive patients that exhibited absent or mild symptoms with no disease progression [632]. Despite detectable viral RNA levels, viral cultures were negative, whole genome sequencing revealed only genomic fragments, and no transmission to contacts was documented by clinical follow-up, acid nucleic tests, and antibody tests. These findings suggested re-detection likely due to intermittent, low-level viral RNA persistence rather than reinfection.

#### Recrudescence

It is important to differentiate between reinfection and recrudescence (i.e., reactivation of lingering virus infection from sanctuary sites). However, this distinction is not straightforward. For example, Gousseff and colleagues reported on a case series of 11 patients with probable reinfection or recrudescence [65]. These individuals were re-positive for SARS-CoV-2 by qRT-PCR, and infectious virus was found in culture swabs from one of only two individuals tested. Reinfection was the likely scenario in a subgroup of younger, healthy HCWs (median age 32 years) that experienced a relatively mild clinical relapse. A durable immune response may have not elicited in these patients because of mild infection. On the contrary, a subset of older patients (median age 73 years) without occupational exposure required hospitalization for both episodes, leading to death in almost half of them. This suggested recurrence, potentially due to suboptimal control of infection, thus allowing a second episode of viral replication.

Viral recrudescence can result in post-discharge positive tests. Recent findings have demonstrated that the Ebola virus can persist in immune-privileged compartments for extended periods following disease recovery [634–637]. Indeed, recrudescence of Ebola virus from the central nervous system has been noted and resulted in viremia and clinical symptoms of disease. Testicular persistence of this virus has also been noted, though viremia or viral detection outside of semen has not been identified in infected patients [638]. While there have been limited investigations of persistence and recrudescence of SARS-CoV-2 in humans, data have emerged, demonstrating that viral components can be found in immune-privileged sites. Yang et al. looked at testes from fatal COVID-19 patients and found significant damage to testicular tissue, including the seminiferous tubules [639]. SARS-CoV-2 RNA was found in <10% of testes sampled and no viral particles were detected by electron microscopy. Ma et al. demonstrated that SARS-CoV-2 can infect testicular cells by revealing in transmission electron microscopy coronavirus-like particles in the interstitial compartment of the testes, in addition to detecting viral RNA [640]. An earlier cohort study from China demonstrated that SARS-CoV-2 RNA could be found in the semen of recovering patients though there were no assessments of infectious virus or longer-term follow-up samples post-discharge [641]. Another investigation noted signs of autoimmune orchitis and impaired spermatogenesis in COVID-19 patients; however, all semen samples in this cohort were negative for SARS-CoV-2 by qRT-PCR [642]. Our investigations of SARS-CoV-2 infections in golden Syrian hamsters have identified viral RNA transiently present within the epididymis and testes of infected animals at two and four days post-infection; however, viral RNA was absent by day seven (Kindrachuk J. et al., unpublished data). Thus, there are inconclusive data at present regarding SARS-CoV-2 persistence within the male reproductive tract.

While brain inflammation and neurological impairment have been recognized in COVID-19 patients, the identification of SARS-CoV-2 in brain tissue remains uncertain [643, 644]. Data from both tissue culture organoids and mouse models suggest that brain tissue can be permissive to infection under these conditions, but the extension of this to natural infection is unknown [645, 646]. Imai and colleagues demonstrated that infectious virus can be recovered from brain samples of infected one-month-old golden Syrian hamsters on day 3 post-infection but absent on day 6 and day 10 [647]. This was found using both high-dose (10^5.6^ plaque-forming units [PFU]) and low-dose (10^3^ PFU) inocula through a combination of intranasal and ocular infection routes. The authors noted that while they demonstrated that SARS-CoV-2 could enter the brain and replicate, viral antigen was not identified in brain tissue.

#### Further research on reinfection and recrudescence is needed

Given the tens of millions of SARS-CoV-2 infections worldwide to date, confirmed reinfections remain an exceedingly rare occurrence. Although rare, publication of reinfections is biased toward the diagnosis of symptomatic cases, with asymptomatic cases likely underreported [598]. Reasons for this are the testing eligibility criteria and the lack of resources and rigorous surveillance in many places, except for routine community testing scenarios such as airports [612] and healthcare settings [617].

In summary, while reinfection and recrudescence appear to be infrequent events, they cannot be dismissed altogether as simple errors or sensitivity issues in current diagnostic technologies. Distinguishing between reinfection and alternative phenomena is not easy and relies on epidemiological analyses (including clinical case history assessment) and virological data (nucleic acid amplification testing and comparative genome analysis) to rule out persistent viral RNA shedding and possibly recrudescence. SARS-CoV-2 reinfection has yet largely unknown implications for immunity. Therefore, further research is warranted.

## Conclusions

This comprehensive narrative review sits at the heart of the science-policy interface, allows an interdisciplinary approach to evidence synthesis, and facilitates step-by-step engagement by readers. We compile and discuss evidence that is useful for decision-makers to consider in the context of a complex policy landscape with many actors and competing priorities and risks. The unfolding pandemic has raised conundrums for which there are no straightforward yes/no answers or unequivocal solutions. False dichotomies are pervasive and attractive—they offer an escape from the unsettling complexity and enduring uncertainty. Besides, faulty reasoning and politicization of uncertainty and disagreement in science preclude debating the merits of various positions and refuting the spurious claims. Uncertainties and complexities are part and parcel of science, public health, and several aspects of pathogen transmission, infection, and disease. These aspects lie on a gradient of gray shades—they are hardly binary, simple, or uniform, and should not be framed as black or white.

Overstated and poor-quality science is harmful and misinforms public health response and policy. In light of the challenges surrounding the science-policy interface for COVID-19, we caution against black-or-white messaging, all-or-nothing guidance, and one-size-fits-all approaches. Subtleties and uncertainties should not be portrayed as enemies but as allies of transparent and accurate messaging, health literacy, critical thinking, and credibility and legitimacy of health authorities. Continued efforts in countering misinformation and disinformation and promoting accuracy in social and mass media are needed.

Public health thrives by providing nuanced guidance that reflects trade-offs and uncertainty, while engaging the public in policy decisions. Culturally appropriate public health communication, science-informed tailored policies, and health journalism that reckon with shades of gray, uncertainties, local contexts, and social determinants are long overdue. As evidence continues to accrue at an unparalleled pace, our understanding of SARS-CoV-2 and COVID-19 evolves allowing policy amendments.

## Data Availability

All studies cited and discussed are peer-reviewed journal articles, preprints, and media articles (gray literature) available in the public domain; some require the relevant database or journal/media subscriptions for access. All data underlying the evidence synthesis of this study are included in the article.

## Abbreviations

AGP: Aerosol-generating procedure
ASI: Asymptomatic SARS-CoV-2 infection
BSL-3: Biosafety level 3
CDC: US Centers for Disease Control and Prevention
COVID-19: Coronavirus disease 2019
Ct: Cycle threshold
FFR: Filtering facepiece respirator
HCW: Healthcare worker
IPC: Infection prevention and control
KDCA: Korea Disease Control and Prevention Agency
MERS: Middle East respiratory syndrome
MIS-A: Multisystem inflammatory syndrome in adults
MIS-C: Multisystem inflammatory syndrome in children
NPI: Non-pharmaceutical intervention
PASC: Post-acute sequelae of COVID-19
PFU: Plaque-forming unit
PM: Particulate matter
PPE: Personal protective equipment
R_0_: Basic reproduction number
RCT: randomized controlled trial
qRT-PCR: quantitative reverse transcriptase-polymerase chain reaction
SARS: Severe acute respiratory syndrome
SARS-CoV-2: Severe acute respiratory syndrome coronavirus 2
SSE: Superspreading event
TBP: Transmission-based precaution
TCID_50_: Median tissue culture infectious dose
WHO: World Health Organization

## Footnotes

1 Infodemic is the overabundance of information (some accurate and some not) making it hard for people to find reliable sources and guidance [2].

2 Broadly, misinformation can be defined as incorrect information, possibly by accident [13]. In contrast, disinformation is often used to denote misinformation that is deliberately false and disseminated.

3 Engagement echo chambers and filter bubbles relate to social media practices that exhibit highly segmented interaction with social media content [14]. Echo chambers may reinforce shared stances and preexisting views of like-minded people. Echo chambers are not the exclusive or necessarily the main mechanism of misinformation.

4 Works deemed essential include but are not limited to healthcare, law enforcement, government, fire department, first responders, delivery/pick-up services, and transportation.

5 A lockdown is understood as a complete shutting down of all economic activity except if deemed most essential, along with stay-at-home orders and usually with stringent travel restrictions [25].

6 Respiratory etiquette or hygiene refers to covering mouth and nose with a disposable tissue when coughing or sneezing and disposing of it after use, or coughing/sneezing into the inner elbows or sleeve, followed by hand hygiene.

7 The term “Emmentaler cheese” is more appropriate than "Swiss cheese" since not all Swiss cheeses have holes [104].

8 SSEs refer to activities and settings where an individual gives rise to a large number of secondary infections [35, 112]. Closed environments, poorly ventilated environments, crowded places, and prolonged exposures all correlate with emergence of SSEs. These hotspots are important as both community infection sources and targets for effective contact tracing, targeted restrictions, and NPIs. SSEs could be influenced by biological features (e.g., an individual with increased infectiousness inferred by viral loads), behavioral/social features (e.g., an infectious individual with high number of susceptible contacts), environmental features (e.g., high-risk settings due to high human interaction or density), and opportunistic features (e.g., singing or activities leading to increased probability of transmission and infection) [35].

9 The dispersion parameter *k* quantifies the variability in the number of secondary cases and is used as a measure of the impact of superspreading. A lower *k* parameter indicates higher transmission heterogeneity (higher superspreading potential). *k* for SARS-CoV-2 has been variably estimated from 0.04 to 0.58 [112–115].

10 COVID-19 absolutism is the discouragement and prohibition of absolutely any behavior leading to some risk of SARS-CoV-2 infection [133].

11 This term has been used to refer to the notion of behavioral fatigue potentially associated with a lower adherence to public health interventions.

12 Outdoor gatherings of household members, sports like tennis, and hiking or jogging while distancing are examples of lower-risk outdoor activities.

13 From a public health perspective, a mass gathering is defined as an event where the number of people attending (usually thousands but this is variable) may strain the planning and response resources of the host community, state, or country [145].

14 Elimination is often defined as the local reduction of infection to zero cases or a prolonged period of zero local transmission in a geographic region, with interventions still required to maintain it [162–164].

15 Eradication is often defined as the global reduction of infection to zero cases as a result of deliberate efforts, with further interventions no longer needed [162–164]. A further and more difficult step is extinction, which is when a pathogen no longer exists in nature or in the laboratory.

16 The incubation period is the time elapsed between infection and symptom onset. It usually ranges from 2 to 14 days. The mean incubation period for SARS-CoV-2 infection is 5.5–6.5 days, and the median incubation period is 5.1 days [118, 166].

17 Secondary attack rate is the percentage of secondary cases (infectees), among the total of contacts, resulting from one infected person (infector).

18 Medical AGPs are procedures that are likely to generate high concentrations of infectious respiratory aerosols (e.g., tracheal intubation, non-invasive positive pressure ventilation), placing HCWs at an increased risk for pathogen exposure and infection [251, 252]. The term AGP is a misnomer inasmuch as it is not the AGP per se that generates aerosols and drives transmission but the circumstances surrounding the AGP, including the air forced over the respiratory mucosa, index patient’s respiratory symptoms, exposure distance, and duration [253].

19 The reproduction number (R) is a measure that reflects the infectiousness of an agent. The basic reproduction number (R_0_) is the average number of secondary infections caused by one infected person in a completely susceptible population, in the absence of interventions. The reproduction number varies according to several circumstances and time (thus called effective reproduction number at time *t*, R_t_) as more people are infected (developing immunity) and public health measures are implemented.

20 Counter-visualizations are counterpublic’s data visualization practices that use orthodox methods to make unorthodox arguments [19]. They may serve the purpose of challenging mainstream narratives and promoting misinformation and conspiracy.

21 The precautionary principle assists with decision-making under uncertainty [525, 526]. Traditionally used to advice caution in the uptake of interventions or innovations with known benefits but uncertain risks [527], the precautionary principle has been substantially used in the opposite sense in the COVID-19 pandemic—to advocate the uptake of interventions because of the potential great benefits and minimal risks [432]. It has been argued that both the omission in the former case and the act in the latter case may prevent harm [528].

22 Masks may cause discomfort and subjective but minor breathing difficulties. However, claims pertaining to decreased oxygen saturation are unfounded. Recent studies assessing medical masks and cloth face coverings in the general adult population have not demonstrated major physiologic changes related to gas exchange [454, 455, 556–561].

23 Acceptable evidence in support of SARS-CoV-2 reinfection consists of testing positive by qRT-PCR at two different time points (usually separated by a period of test negativity if documented), plus distinct genome sequencing of viral isolates on each episode [62, 63]. The lack of these data, which is expected outside research settings, hinders reporting of reinfection. In regions with robust genomic surveillance, sequencing a SARS-CoV-2 genetic variant from a sample of the second infection episode provides evidence of reinfection if the variant was not present in the region at the time of the first episode [623].

24 Re-positive or recurrent-positive cases are individuals who recovered from previously confirmed COVID-19 disease and had a positive test again, attributed to the same infection episode.
